# Transdiagnostic reduction in cortical choline-containing compounds in anxiety disorders: a ^1^H-magnetic resonance spectroscopy meta-analysis

**DOI:** 10.1038/s41380-025-03206-7

**Published:** 2025-09-05

**Authors:** Richard J. Maddock, Jason Smucny

**Affiliations:** Department of Psychiatry and Behavioral Sciences, University of California, Davis, CA USA

**Keywords:** Psychiatric disorders, Neuroscience

## Abstract

**Background:**

Anxiety disorders (AnxDs) are highly prevalent and often untreated or unresponsive to treatment. Although proton magnetic resonance spectroscopy (1H-MRS) studies of AnxDs have been conducted for over 25 years, a consensus regarding neurometabolic abnormalities in these conditions is lacking.

**Methods:**

A systematic review and meta-analysis of 1H-MRS studies of AnxDs (social anxiety disorder, generalized anxiety disorder, and panic disorder) identified 25 published datasets meeting inclusion criteria. These compared neurometabolites between 370 patients and 342 controls, including n-acetlyaspartate (NAA), total creatine, total choline (tCho), myo-inositol, glutamate, glutamate+glutamine, GABA, and lactate.

**Results:**

Across AnxDs, tCho was significantly reduced in prefrontal cortex and across all cortical regions. Effect sizes for cortical tCho were significantly more negative in studies with better measurement quality, with Hedges’ *g* = −0.64 and an 8% mean reduction. NAA was unchanged in prefrontal cortex but reduced across all cortical regions (after exclusions). These abnormalities did not differ between the three disorders. No other neurometabolites differed significantly.

**Discussion:**

Reduced choline-containing compounds in cortical regions is a consistent, transdiagnostic abnormality in AnxDs. Notably, arousal-related neuromodulators, including norepinephrine, alter membrane phospholipid homeostasis and methylation reactions, which influence brain tCho levels. This suggests that chronically elevated arousal in AnxDs may increase neurometabolic demand for choline compounds without a proportionate increase in brain uptake, leading to reduced tCho levels. Reduced cortical NAA suggests compromised neuronal function in AnxDs. Future studies may clarify the clinical significance of reduced cortical tCho and the possibility that appropriate choline supplementation could have therapeutic benefit in anxiety disorders.

## Introduction

Anxiety disorders (AnxDs), including generalized anxiety disorder (GAD), panic disorder (PD), and social anxiety disorder (SAD), are common, debilitating maladies that affect around 30% of United States adults at some point in their lives, making it the most common category of mental illness [1]. Treatments with proven efficacy for these disorders include psychotherapies and pharmacotherapies [2]. However, approximately one-third of patients needing treatment may not receive treatment [3], and 35–60% of treated patients may not achieve clinical remission [4].

A more complete understanding of the neurobiological underpinnings of AnxDs will help guide the development of more effective interventions. The neural mechanisms of vulnerability to AnxDs can be broadly conceptualized in terms of two distinct, but not mutually exclusive, processes. One process implicates a relative incapacity of prefrontal executive control circuits to govern limbic and brainstem threat and arousal reactions [5, 6]. In this scheme, vulnerability could originate from impaired function of executive circuits and/or abnormally heightened reactivity of threat and arousal circuits [7]. A second process can occur when prefrontal executive processes maladaptively amplify the reactivity of lower threat and arousal circuits [8, 9]. In this scheme, both executive and threat/arousal circuits are functioning normally at the biological level, but their feedback interactions produce a vicious cycle of anxiety and distorted cognitions. Whatever the underlying causes, AnxDs are transdiagnostically associated with chronically or recurrently elevated anxiety and arousal [10, 11] with attendant neurobiological adaptations that may themselves have pathogenic consequences.

Proton magnetic resonance spectroscopy (1H-MRS) studies of the brain can potentially identify neurometabolic signs of abnormal neural functioning in AnxDs. The most commonly measured brain metabolites in AnxD studies include n-acetylaspartate (NAA), total choline-containing compounds (tCho), and creatine plus phosphocreatine (tCr). NAA is produced by neuronal mitochondria and catabolized in oligodendrocytes where it contributes acetate moieties for myelin synthesis [12]. It is considered an indicator of neuronal integrity. The tCho signal is comprised mainly of glycerophosphocholine and phosphocholine [13]. Both are soluble intermediates in the metabolism of membrane phospholipids. Abnormal tCho is generally viewed as reflecting changes in membrane dynamics or phospholipid metabolism such as those occurring with aging, gliomas, and endocrine disorders [14–16]. Together, creatine and phosphocreatine are essential for energy metabolism in all brain cells [17]. Although their ratio varies with energy status, their sum is thought to be relatively stable in healthy volunteers [18]. Abnormal creatine levels have been reported in some psychiatric disorders compared to age-matched controls [19, 20]. However, such reports have generally not been confirmed at the meta-analytic level [21, 22] with the exception of reduced levels in Alzheimer’s disease and autism [23, 24].

Less commonly reported metabolites in AnxD studies include myo-inositol, glutamate, GABA, and lactate. Myo-inositol functions in osmotic regulation [25] and has been associated with neuroinflammation, glial activation, and gliosis [26, 27]. Glutamate is the primary excitatory neurotransmitter and also participates in the tricarboxylic acid cycle [28]. The combined signal from glutamate plus glutamine (glx) is often reported in 1H-MRS studies [17]. Detection of GABA, the primary inhibitory neurotransmitter, requires specialized acquisition sequences [29]. Reduced GABA is often interpreted as evidence of a reduced capacity for inhibitory neurotransmission. Lactate can be reliably measured using optimized acquisitions [17]. Elevated lactate can reflect hypoxia or increased aerobic glycolysis under normoxic conditions [30].

Since the 1990, many investigators have published brain 1H-MRS studies of people with SAD, GAD, and PD. Although specific neurometabolic abnormalities have been reported, many inconsistencies have also been observed. One replicated finding has been reduced tCho in various cortical regions in AnxDs [31–33], a finding opposite to the elevated tCho seen in other psychiatric disorders such as schizophrenia, bipolar disorder and major depression [21, 22, 34]. A consistent pattern of regional neurometabolic abnormalities in AnxDs might implicate prefrontal executive regions, limbic or brainstem regions, and/or a widespread neurometabolic disturbance as important pathophysiological features. The current study is the first meta-analysis to examine this literature for consistent neurometabolic abnormalities in AnxDs. As an index of neuronal integrity, we hypothesized reduced NAA in prefrontal regions in AnxDs implicating impairment of executive control. We also hypothesized abnormal tCho across multiple regions in AnxDs, implicating widespread neurometabolic changes in these disorders.

## Methods

### Study selection

For systematic review, the PubMed database was searched on September 25, 2024, to identify journal articles using the following query: ((*anxiety disorder OR social anxiety disorder* OR *panic disorder*) AND (*magnetic resonance spectroscopy* OR *magnetic resonance spectroscopic* OR ^*1*^*H-MRS*)). This search yielded 443 records for screening. Records were screened manually by both authors with discrepancies discussed and resolved. Records not full text, English-language, brain 1H-MRS studies comparing patients currently meeting diagnostic criteria for GAD, PD, or SAD to healthy controls (HCs) were excluded. Thirty-four remaining records were retrieved and further examined for eligibility (PRISMA diagram, Fig. S1).

### Data extraction

For each brain region, author J.S. extracted and R.J.M. verified data. Extracted data included diagnosis (SAD, GAD, or PD), sample sizes, means, and standard deviations (SDs) for each metabolite. Three authors were contacted to provide mean and SD data not included in the published papers [30, 35, 36]. All three provided the missing information. Field strength, echo time (TE), metabolite normalization method, percent of participants of each sex, mean ages, percents of patients taking antidepressants and benzodiazepines, comorbidities, and spectral quality metrics (CRLB, linewidth, and signal-to-noise ratio) were also extracted.

### Eligibility criteria

Two datasets reporting on remitted patients were excluded [36, 37]. When studies reported partially overlapping samples, only the dataset with the largest sample size was included (4 excluded) [38–41]. When metabolite values normalized to both water and tCr were reported [42–45], the normalization producing the lowest coefficient of variation (COV) averaged across groups was used [46]. Data reported only as signal-to-noise values were excluded [45]. One GABA dataset not using spectral editing [47], and two lactate datasets not using editing or optimal TE [31, 47] were excluded.

### Data analysis

Regional metabolite effect sizes for each dataset were calculated as Hedges’ *g* [48]. Meta-analyses were performed only if ≥ three datasets were available. With our a priori interest in widespread neurometabolic effects of chronic anxiety, we conducted a general cortical meta-analysis in addition to regional meta-analyses. We defined “cortical” as regions primarily comprised of either cortical gray matter, white matter directly associated with cortical gray matter, or a mixture of both. When studies reported on multiple cortical regions, only the region with the lowest mean COV was included in the cortical meta-analysis.

For meta-analyses, the significances of both pooled effect sizes and residual heterogeneities were calculated with the Cochran Q test using inverse variance-weighted, random effects models and the restricted maximum likelihood method. The analyses were conducted with JASP software (version 0.19.1), which uses the R-based metafor package as its computational engine [49]. Heterogeneity across studies was also quantified as I^2^. Small sample study bias was examined using Egger’s regression test for funnel plot asymmetry. A significant Egger’s test (*p* < 0.05) would be followed by re-analysis with the most biasing dataset removed. When the 95% confidence interval (CI-95) of the metabolite effect size from an individual dataset did not overlap with the CI-95 for the meta-analysis in which it was included, that metabolite value was considered a statistical outlier, and the meta-analysis was repeated with that value excluded.

For metabolites found to be significantly different between patients and controls in any contrast, we calculated the patient versus control weighted mean percent difference across datasets for that metabolite in all associated contrasts, as follows:

$$\frac{{\sum }_{i=1}^{k}[({{\rm{pt}}}{\bar{x}}_{i}-{{\rm{con}}}{\bar{x}}_{i})/({{\rm{con}}}{\bar{x}}_{i})]\ast ({{\rm{pt}}}{N}_{i}+{{\rm{con}}}{N}_{i})}{{\sum }_{i=1}^{k}{{\rm{pt}}}{N}_{i}+{{\rm{con}}}{N}_{i}}$$

where k = the number of datasets, ${{\rm{pt}}}\bar{{x}_{i}}$ and ${{\rm{con}}}\bar{{x}_{i}}$ = mean patient and control metabolite value in each dataset, pt *N*_*i*_ and con *N*_*i*_ = number of patients and controls in each dataset.

### Moderator analyses

The influence of clinical and technical factors on regional metabolite differences was examined by moderator analyses. Differences attributable to specific diagnoses (SAD vs. GAD vs. PD), normalization method (water versus tCr), and field strength (<3 T versus ≥3 T) were analyzed by subgroup analyses. Subgroups were also created for patient medication status based on the observed distribution of this variable (see Results). Moderating effects of mean patient age, percent of male patients, and log TE were examined by meta-regression.

As previously demonstrated [21, 46], we anticipated that true group differences in metabolite values would be most evident in studies with better metabolite measurement quality, with pooled effect sizes becoming larger and more consistent above an identifiable measurement quality threshold. The method of calculating the significance of the association between measurement quality and pooled effect size is detailed in the Supplemental Methods and prior publications [21, 46]. Candidate quality metrics included linewidth, signal-to-noise ratio, CRLB, and coefficient of variation (COV, calculated as the ratio of the SD to the mean) of metabolite values (averaged across patient and control groups). As in prior meta-analyses, this moderator analysis required a minimum of 14 datasets [46]. Other subgroup and meta-regression analyses required at least 10 total datasets, with at least 2 datasets per subgroup for subgroup comparisons [50, 51].

### Study quality assessment

We assessed study quality and risk of bias in all included datasets. For this, we adapted the checklist of Das et al. [52] to create a 14 item scale with 7 items addressing sample characteristics and 7 items addressing MRS methodology (Table S3).

## Results

### Overview

Of 34 articles assessed for eligibility (Fig. S1), data from 25 datasets from 24 articles were extracted for inclusion in meta-analyses [30–33, 35–37, 42–45, 47, 53–65] (Table S1). Summary information is provided in Table S2. Briefly, mean patient age ranged from 22 to 45 years (study average = 35) with a slight preponderance of female patients (study average = 61%). Eleven datasets reported on PD, nine on GAD, and six on SAD. One article stated patients had a lifetime GAD diagnosis, without specifying whether they currently met diagnostic criteria [35]. All statistical analyses were conducted with and without this dataset included. Twenty of the 25 datasets included only unmedicated patients. Twenty-one reported sufficient information about current comorbidity to be classified as “none,” (k = 11), “minor” (k = 4) or “substantial” (>25% of sample) (k = 6) (Table S4). Comorbidity was highest in GAD and lowest in SAD datasets. The most common comorbid conditions were other anxiety disorders, followed by dysthymia and major depression. Field strength ranged from 1.5 to 4 Tesla, with 14 datasets <3 T and 11 ≥ 3 T. Seven of the 25 datasets used multivoxel acquisitions and one reported both single and multivoxel data. Twenty used PRESS and five used STEAM sequences for localization.

### Patient vs. control differences

Results of all primary meta-analyses comparing AnxD patients to HCs are summarized in Table 1.

**Table 1:** Primary meta-analyses of neurometabolite levels in anxiety disorders.

	Region	K	Pts	HC	Effect Size (95% CI)	P value	Percent Diff^a^ Pt - HC	Heterogeneity I^2%^, Q, P value
***tCho***	ACC	7	109	112	+0.01 (−0.35 to +0.36)	0.96	+0.7%	41.0, 10.4, 0.11
	**PFC**^b^	**6**	**78**	**89**	−**0.46** (−**0.15 to** −**0.77)**	**0.0035**	−**7.5%**	0.0, 2.7, 0.74
	**All Cortical**^c^	**16**	**234**	**244**	−**0.30** (−**0.01 to** −**0.58)**	**0.041**	−**4.2%**	57.2, 35.2, 0.002
	**Excl. outliers**^**d**^	**14**	**193**	**209**	−**0.45** (−**0.25 to** −**0.65)**	**0.00001**	−**6.9%**	0.0, 12.2, 0.51
	Basal ganglia^e^	4	65	68	−0.17 (+0.17 to −0.51)	0.32	−3.4%	0.0, 2.0, 0.58
	Hippocampus^b^	3	51	45	+0.14 (−0.27 to +0.54)	0.51	+1.5%	0.0, 2.0, 0.36
***NAA***	ACC	7	108	111	+0.12 (−0.41 to +0.66)	0.65	+0.4%	72.7 23.3 0.0007
	PFC^b^	7	93	104	−0.07 (−0.45 to +0.32)	0.74	+0.1%	43.5, 10.6, 0.10
	Occipital	3	33	37	−0.47 (+0.01 to −0.95)	0.055	−4.3%	0.0, 0.56, 0.76
	White matter	3	54	42	−0.33 (+0.09 to −0.74)	0.12	−5.2%	0.0, 1.6, 0.45
	All Cortical^c^	18	269	275	−0.11 (+0.15 to −0.37)	0.41	−1.7%	56.3, 39.3, 0.002
	**Excl. outliers**^**d**^	**16**	**232**	**238**	−**0.26** (−**0.08 to** −**0.45)**	**0.005**	−**3.5%**	0.0, 12.1, 0.67
	Basal ganglia^e^	5	84	87	−0.20 (+0.19 to −0.60)	0.31	−3.8%	38.9, 7.0, 0.13
	Hippocampus^b^	4	65	52	+0.01 (−0.36 to +0.38)	0.96	−0.5%	0.0, 1.6, 0.65
***tCr***	ACC	4	58	60	+0.05 (−0.32 to +0.41)	0.81		0.0, 1.1, 0.77
	PFC	4	46	57	−0.18 (+0.41 to −0.78)	0.54		54.6, 6.5, 0.09
	All Cortical	9	118	135	−0.15 (+0.13 to −0.44)	0.30		20.2, 10.8, 0.22
**m-Inositol**	ACC	4	77	77	−0.18 (+0.14 to −0.50)	0.26		0.0, 1.0, 0.81
	PFC	3	36	47	−0.15 (+0.38 to −0.68)	0.57		30.1, 2.9, 0.24
	All Cortical	8	132	134	−0.11 (+0.13 to −0.37)	0.37		0.0, 5.6, 0.58
**Glutamate**	ACC	3	38	38	+0.16 (−0.91 to +1.23)	0.76		80.0, 10.7, 0.005
	All Cortical	5	59	70	−0.22 (−0.69 to +0.25)	0.36		42.3, 6.9, 0.14
**Glx**	ACC	3	52	53	−0.06 (+0.48 to −0.60)	0.82		46.5, 3.7, 0.16
	All Cortical	6	92	97	−0.11 (+0.23 to −0.45)	0.52		27.1, 6.6, 0.25
**GABA**	All Cortical	3	42	39	−0.26 (+0.53 to −1.04)	0.52		66.3, 6.1, 0.048
**Lactate**	All Cortical	3	33	37	+0.04 (+0.52 to −0.44)	0.88		2.1, 2.3, 0.31

^a^Weighted mean percent difference between patients and controls. See Methods.

^b^For PFC and HC, right was studied more often than left, so right was included when both right and left were reported.

^c^When more than one cortical region was reported, the region with the lowest mean COV was retained.

^d^After exclusion of datasets with an effect size that fell outside the 95% confidence limits for this analysis.

^e^For basal ganglia, left was studied more often than right, and putamen more often than caudate. Left putamen was preferentially included when it was reported.

*K* number of datasets, *Pts* patients, *HC* healthy controls, *CI* confidence interval, *tCho* total choline-containing compounds, *NAA* n-acetylaspartate, *tCr* creatine+phosphocreatine, *Glx* glutamate+glutamine, *ACC* anterior cingulate cortex, *PFC* prefrontal cortex.

Rows with bolded values indicate statistically significant effects.

### Cortical regions

#### Choline-containing compounds (tCho)

**Fig. 1:**
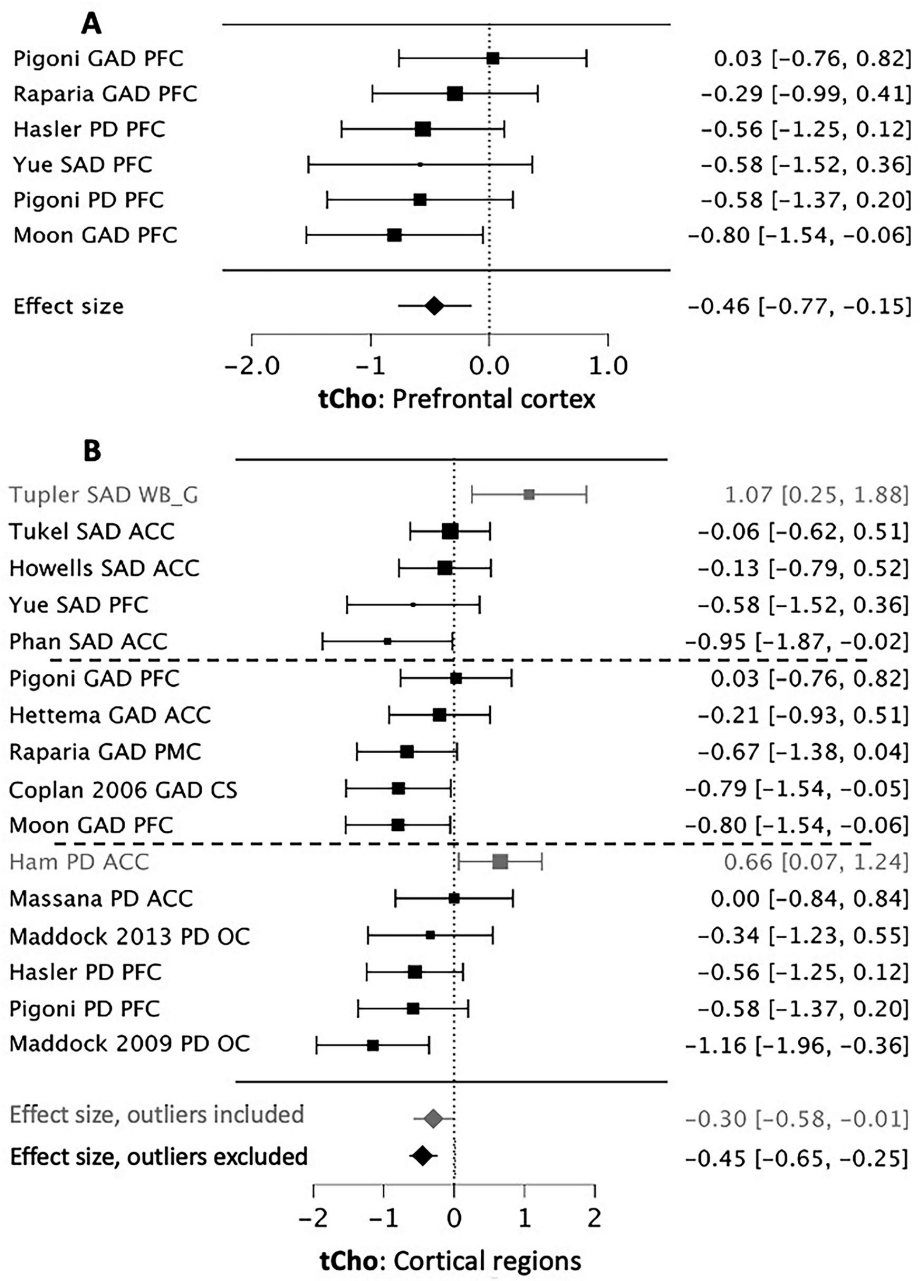
Forest plots for significantly reduced tCho in anxiety disorders. First author, diagnosis, and brain region are at left. Year is included if more than one first author paper by the same author is cited. Hedges’ *g* and 95% CI are at center and right. Effect sizes < zero indicate lower levels in the AnxD group. **A**. Six datasets reporting tCho from prefrontal cortex. When bilateral PFC regions were reported, the most commonly reported side (right) was retained for analysis. **B**. Sixteen datasets reporting tCho levels from any cortical region. When multiple cortical regions were reported, the region with the lowest coefficient of variation for tCho values (averaged over patients and controls) was retained for analysis. Horizontal dotted lines separate specific AnxD diagnoses. Gray text indicates outlier datasets, defined as having 95% confidence intervals (CIs) that do not overlap the 95% CIs for the overall meta-analysis. ACC anterior cingulate cortex, CS centrum semiovale, PFC prefrontal cortex, PMC premotor cortex, OC occipital cortex, WB_G whole brain gray matter.

The anterior cingulate cortex (ACC) was the most commonly studied region (7 datasets, 109 Pts, 112 HCs) [32, 35, 42, 43, 47, 53, 54]. It showed no significant difference in tCho between AnxDs and HCs (*g* = +0.01, 95% CI = −0.35 to +0.36, p = 0.96). Meta-analysis of six prefrontal cortex (PFC) datasets (78 Pts, 89 HCs) [31, 42, 58, 59, 61] showed significantly reduced tCho in AnxDs compared to HCs (*g* = −0.46, 95% CI = −0.77 to −0.15, p = 0.003) (Fig. 1a). Less than three datasets reported tCho data from occipital, parietal, temporal or insular cortical regions. Sixteen datasets reported on tCho measured in at least one cortical region (defined above). Seven of these reported on multiple cortical regions [32, 33, 42, 54, 55, 59, 61]. Only one cortical region per dataset was included, based on having a lower coefficient of variation (COV) for tCho measurements as a proxy for measurement quality [21, 46]. The analyzed regions included 6 ACC [32, 35, 43, 47, 53, 54], 5 PFC [31, 42, 58, 61], 2 occipital [30, 36], 1 premotor [59], 1 centrum semiovale [33], and 1 whole brain cortical gray matter [55]. Meta-analysis across these regions (234 Pts, 244 HCs) showed significantly lower cortical tCho levels in the patient group (*g* = −0.31, 95% CI = −0.01 to −0.60, p = 0.040). Two datasets were identified as outliers, as their 95% CIs did not overlap the overall 95% CI (one whole brain gray cortical matter and one ACC). Repeating the analysis with these excluded (193 Pts, 209 HCs) demonstrated a larger and more consistent decrease in cortical tCho in AnxDs (*g* = −0.46, 95% CI = −0.25 to −0.67, p < 0.00001) (Fig. 1b).

#### N-acetylaspartate (NAA)

**Fig. 2:**
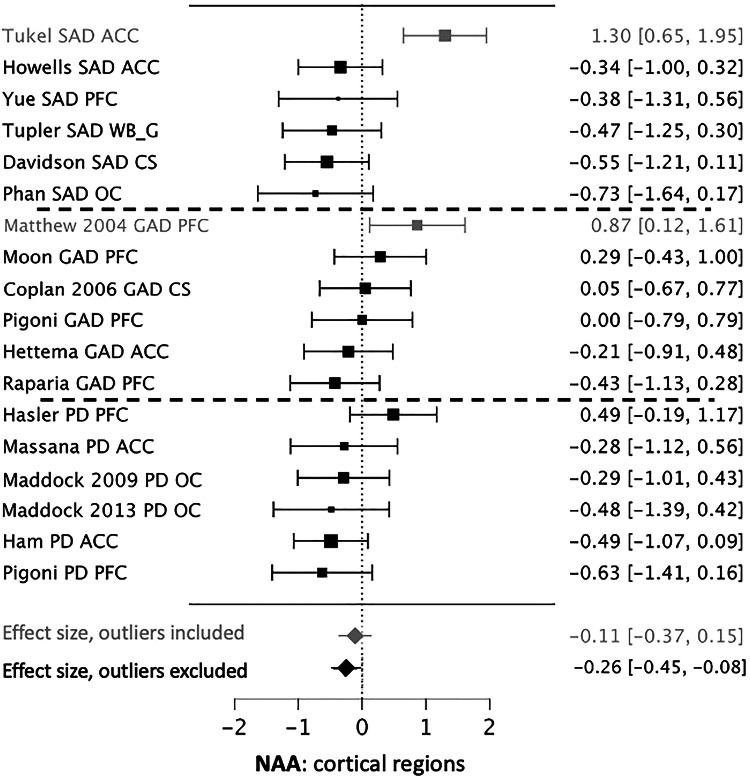
Forest plot for reduced cortical NAA in anxiety disorders. Descriptions and abbreviations are the same as in Fig. 1.

Seven datasets reporting NAA were available for both the ACC [32, 35, 42, 43, 47, 53, 54] and the PFC [31, 42, 56, 58, 59, 61]. No significant or trend level changes were observed in either region. Three datasets reported NAA in the occipital cortex [30, 32, 36]. These showed a trend-level reduction in NAA in AnxDs (*g* = −0.47, 95% CI = +0.01 to −0.95, p = 0.055). Three datasets reporting NAA in white matter regions showed no significant effect [33, 45, 55]. Less than 3 datasets reported NAA data from parietal, temporal or insular cortical regions. Eighteen datasets reported NAA measured in at least one cortical region [30–33, 35, 36, 42, 43, 45, 47, 53–56, 58, 59, 61]. Seven datasets reported on more than one cortical region [32, 33, 42, 54, 55, 59, 61]. For this analysis, we included only one region per dataset based on having the lowest COV for NAA. The analyzed regions included 5 ACC [35, 43, 47, 53, 54], 7 PFC [31, 42, 56, 58, 59, 61], 3 occipital [30, 32, 36], 2 centrum semiovale [33, 45], and 1 whole brain cortical gray matter [55]. No significant difference in NAA levels was seen across these regions, but two datasets were identified as outliers (1 ACC and 1 PFC). Repeating the analysis with these excluded demonstrated a significant decrease in cortical NAA in AnxDs (232 Pts, 238 HCs, *g* = −0.26, 95% CI = −0.45 to −0.08, p < 0.005) (Fig. 2).

#### Other cortical metabolites

No significant differences between patient and controls were observed for any other metabolites in cortical regions. These analyses for tCr [32, 33, 35, 36, 42, 47, 58, 59], myo-inositol [31, 35, 47, 53–55, 58], glutamate [32, 44, 53, 58], glx [31, 35, 47, 61], GABA [61–63] and lactate [30, 36, 64] are summarized in Table 1 and detailed in the Supplemental Figures and Results.

### Subcortical regions

No significant differences between patient and controls were observed for tCho or NAA in the basal ganglia [42, 45, 47, 53, 54] or hippocampus [35, 43, 57, 60]. These analyses are summarized in Table 1 and in the Supplemental Figures. No other subcortical regional metabolites were reported by k ≥ 3 datasets.

All above cortical and subcortical analyses showed the same pattern of statistical results when confined to studies using single voxel methods.

### Moderating factors

Factors potentially moderating patient versus control differences were examined if regional metabolites were reported by at least 10 datasets (14 for measurement quality). The only regional metabolites meeting these criteria were cortical tCho and cortical NAA. Potentially moderating effects of specific diagnosis, metabolite measurement quality, medication status, normalization method (tCr vs. water), percent male patients, mean patient age, log TE, field strength, and study quality were examined for these cortical metabolites.

#### Moderating factors - anxiety disorder diagnosis

Results of analyses of the moderating effect of diagnosis and patient versus control differences within specific diagnoses are summarized in Table S5.

#### Choline-containing compounds

Of the 16 datasets reporting cortical tCho levels, five studied patients with SAD, six studied patients with PD and five studied patients with GAD. Diagnosis had no significant effect on patient versus control differences in tCho level for cortical regions (p = 0.58). When excluding two outlier tCho datasets identified in the primary meta-analysis of cortical regions (“primary outliers”) (Fig. 1b), the effect of diagnosis remained non-significant (p = 0.57). When each diagnosis was considered separately, only GAD showed significantly reduced tCho (k = 5, 73 Pts, 79 HCs, *g* = −0.50, 95% CI = −0.17 to −0.83, p = 0.003) (Table S5). When excluding the primary outlier datasets (one PD and one SAD), PD showed significantly reduced tCho (k = 5, *g* = −0.55, p = 0.002) and the pooled effect size in SAD become more negative (k = 4, *g* = −0.29, p = 0.11) (Table S5). There was no evidence of a significant difference in cortical tCho effect sizes between the specific AnxD diagnoses.

#### N-acetylaspartate

Of the 18 datasets reporting cortical NAA levels, six studied SAD, six studied PD and six studied GAD. Diagnosis had no significant effect on patient versus control differences in NAA (p = 0.56). This was unchanged after excluding the primary outliers (p = 0.23). When analyzing each diagnosis subgroup separately, no significant effect on cortical NAA was seen for any specific diagnosis. After excluding primary outliers, NAA was significantly reduced in patients with SAD (k = 5, 76 Pts, 64 HCs, *g* = −0.48, 95% CI = −0.14 to −0.82, p = 0.006), but remained non-significant in GAD and PD (Table S5). Since removing one extreme statistical outlier [54] had a substantial impact on the results for SAD, possible reasons for the outlier value were examined. This revealed evidence of possible error in the reported NAA mean and/or SD values, as described in Supplemental Results. We contacted the author requesting clarification but received no response.

#### Other metabolites

No other metabolites had sufficient datasets for comparison of effect sizes between specific AnxD diagnoses. Meta-analyses within specific diagnoses for other metabolites with at least 3 datasets in a diagnosis group are described in Table S5 and in the Supplemental Results. No significant differences were observed in these metabolites for any specific AnxD diagnoses.

#### Moderating factors - metabolite measurement quality

Insufficient datasets reporting CRLB, linewidth, or signal-to-noise ratio for any regional metabolite were available to examine the influence of measurement quality using these metrics. Mean regional metabolite COV was available for all datasets. The number of datasets was ≥14 for only cortical tCho and cortical NAA. Analyses of the moderating effect of COV as a proxy for metabolite measurement quality are summarized in Table 2.

**Table 2:** Moderation of cortical metabolite effects by coefficient of variation (COV) as a quality metric.

	Subgroups	K	Pts	HC	Effect Size with (95% CI), or r^2^ and IP	P value	Percent Diff Pt - HC	Heterogeneity I^2^% P value
**Cortical tCho**	**COV logistic fit**	**16**	**234**	**244**	**r**^**2**^ = **0.61, IP (*g*)** = −**0.31**	**0.035**	n/a	n/a
	**COV logistic fit** **excl 1° outliers**	**14**	**193**	**209**	**r**^**2**^ = **0.96, IP (*g*)** = −**0.48**	**0.00086**	n/a	n/a
	Lo vs Hi COV^a^	14	193	209	Lower tCho with <COV	**0.05**	n/a	0.0, 8.4, 0.76
	**COV** ≤ **17%**^a^	**8**	**100**	**116**	−**0.64 (**−**0.36 to** −**0.92)**	**<0.00001**	−**8.0%**	0.0, 5.6, 0.58
	COV ≥ 19%^a^	6	93	93	−0.24 (+0.05 to −0.53)	0.10	−5.5%	0.0, 2.7, 0.74
**Cortical** **NAA**	COV logistic fit	18	269	275	r^2^ = 0.05, IP = n/a	0.37	n/a	n/a
	COV logistic fit excl 1° outliers	16	232	238	r^2^ = 0.18, IP = n/a	0.27	n/a	n/a

^a^Low and high COV datasets defined based on COV at the inflection point of the logistic curve. See Methods.

*COV* coefficient of variation of measured metabolite values, calculated as the unweighted mean of the COVs of patient and control participants for each dataset, *IP* empirical inflection point of logistic curve in effect size units; excl 1° outliers, after exclusion of datasets with an effect size that fell outside the 95% confidence limits for the primary meta-analysis of patient versus control differences, as shown in Table 1. Other abbreviations as in Table 1.

Rows with bolded values indicate statistically significant effects.

#### Choline-containing compounds

**Fig. 3:**
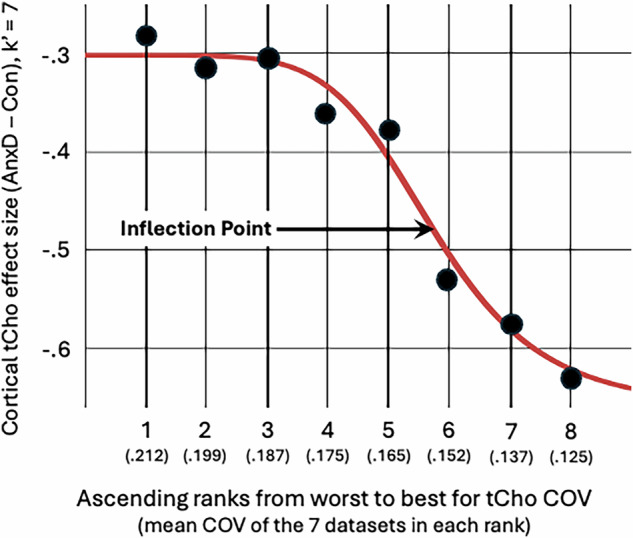
Stronger evidence for reduced cortical tCho in datasets with better measurement quality. Black circles represent the moving sample pooled effect size from 7 cortical datasets in ascending ranks of coefficients of variation (COVs) from poorer (left) to better COV (right) along the x-axis. Circle #1 is the effect size for seven datasets ranked 8–14. Circle #8 is for datasets ranked 1–7 (total k = 14). Mean COV for each set of 7 studies is given in parentheses along the x-axis. Red line is best-fitting logistic function. Inflection point is empirical threshold separating low- and high-quality datasets.

The moderating effect of mean COV on the patient versus control effect sizes for cortical tCho was examined across 16 datasets. The best-fitting logistic fit between Hedge’s *g* values from successive subsamples of k′ = 7 datasets ranked by COV quality and the corresponding COV quality ranks was significant (r^2^ = 0.61, p = 0.035), with an inflection point at *g* = −0.31 (Table 2). When the 2 outliers from the primary analysis of cortical tCho were excluded, the logistic fit was even more striking (r^2^ = 0.96, p < 0.001), with an inflection point at *g* = −0.48 (Table 2). The logistic curve illustrating this relationship is shown in Fig. 3. The inflection point empirically divides the datasets into 6 lower measurement quality (COV = 19%–24%, mean = 22%) and 8 higher measurement quality (COV = 8–17%, mean = 13%) subgroups. Direct comparison of the subgroups showed a significantly greater reduction in cortical tCho in AnxD patients from studies with higher quality tCho measurements (Table 2).

#### N-Acetylaspartate

The moderating effect of mean COV on the patient versus control effect sizes for cortical NAA was examined across 18 datasets. The best-fitting logistic fit between the k′ = 7 pooled sub-meta-analytic effect sizes and the COV quality ranks was not significant, either across all 18 datasets (r^2^ = 0.05, p = 0.37) or across 16 datasets after excluding the 2 outliers from the primary analysis of cortical NAA (r^2^ = 0.18, p = 0.27).

#### Moderating factors - medication status

Twenty datasets included 100% unmedicated patients. The remaining 5 datasets included some patients taking psychiatric medications. The percent of patients taking antidepressants in the latter datasets ranged from 52 to >90% (Table S2). In four of these five datasets, 24 to >90% of patients were also taking benzodiazepines. When confining our analyses to datasets reporting on unmedicated patients only, the pattern of results mirrored that shown in Table 1 for all metabolites except tCr (Table S6). Cortical tCr was significantly reduced in patients in unmedicated datasets (k = 5, 58 Pts, 62 HCs, *g* = −0.50, 95% CI = −0.14 to −0.87, p = 0.007), an effect not observed in the analysis across all datasets. For cortical tCho and cortical NAA, patient versus control effect sizes were similar in unmedicated and medicated datasets, with or without primary outliers (Table S6). Similarly, for all other metabolites, the results from unmedicated datasets mirrored the overall non-significant results.

#### Moderating factors - normalization method, sex, age, field strength, and echo time

Seventeen datasets reported tCr-normalized metabolites and nine reported water-normalized metabolites (including four that reported both). Four others normalized with phantom replacement and one reported only lactate normalized to NAA. Group-wise contrasts comparing patient versus control effect sizes when normalizing to creatine versus normalizing to water were not significant for either cortical tCho or cortical NAA. Similarly, sub-group comparisons of magnetic field strength < 3 T versus ≥ 3 T and meta-regressions using either percent of male patients, mean patient age, or log TE as the regressor all showed no significant effect on effect sizes for cortical tCho or NAA (Table S7). The same pattern of results was seen when primary outliers were excluded from these moderator analyses.

### Study quality, publication bias, and heterogeneity

Study quality ratings ranged from 9–14 on a 14-point scale (median = 11) (Tables S2 and S3). Quality ratings did not differ among the three diagnoses but were anti-correlated with COV for cortical tCho (r = −0.61, p = 0.02, n = 14) with primary outliers excluded. Higher quality studies had significantly lower COVs for cortical tCho. Study quality significantly moderated the effect size for cortical tCho with outliers included, showing more reduced tCho in studies with higher quality ratings. This effect was not significant after excluding outliers (Table S7). Study quality did not correlate with NAA COV or significantly moderate the effect size for cortical NAA (Table S7). Egger’s test for funnel plot asymmetry suggesting small study bias was not significant in any of the primary or moderator meta-analyses described above. Significant heterogeneity (Q statistic, p < 0.05) was observed in the primary meta-analyses for both cortical tCho and cortical NAA. None of the moderating factors accounted for this heterogeneity, which remained significant in all moderator analyses. Instead, statistically outlying cortical tCho and NAA datasets (two each) appeared to account for it, as heterogeneity was low (I^2^ < 25%) and non-significant (p >0.2) in all primary and moderator analyses of cortical tCho and NAA when the outliers were excluded. Similarly, significant heterogeneity in the ACC-NAA meta-analysis became non-significant (p = 0.21) when a primary cortical outlier was removed (I^2^ = 21%). Heterogeneity was non-significant in all other meta-analyses, except for ACC glutamate (k = 3) and cortical GABA (k = 3), in which instances no clear cause was evident.

All of the reported analyses exhibited the same pattern of statistical significance when excluding the study that reported only lifetime diagnoses [35].

## Discussion

The primary finding of this first 1H-MRS meta-analysis in AnxDs is that tCho levels were significantly reduced in the PFC region and across cortical tissues generally in these disorders. These reductions were significant with or without excluding outlier datasets. The moderation analysis revealed that pooled effect sizes for reduced cortical tCho were greater across studies with better metabolite measurement quality, further validating the primary finding. A secondary finding was that NAA was significantly reduced across cortical regions after excluding outlier datasets. The same patterns of tCho and NAA results were seen when the analyses were restricted to unmedicated datasets, nor did they differ when comparing mostly medicated to unmedicated datasets. No significant effects were observed in the primary meta-analyses of other metabolites, nor did metabolite effects differ significantly between the three AnxD diagnoses. Metabolite effects were not moderated by patient age, percent male, normalization method, field strength, or echo time.

### Reduced tCho

Reduced tCho in AnxDs contrasts with elevated tCho reported in meta-analyses of some other neuropsychiatric disorders and conditions, including schizophrenia, bipolar disorder, traumatic brain injury, and HIV infection [21, 34, 66–68]. In these reports, elevated tCho has been interpreted as evidence of membrane or myelin disruption, increased membrane turnover, neuroinflammation, or increased cell density. While reduced tCho is less commonly reported in neuropsychiatric conditions, examination of metabolic processes that influence tCho levels and conditions characterized by reduced brain tCho may shed light on the possible pathophysiological and clinical significance of reduced tCho in ADs.

**Fig. 4:**
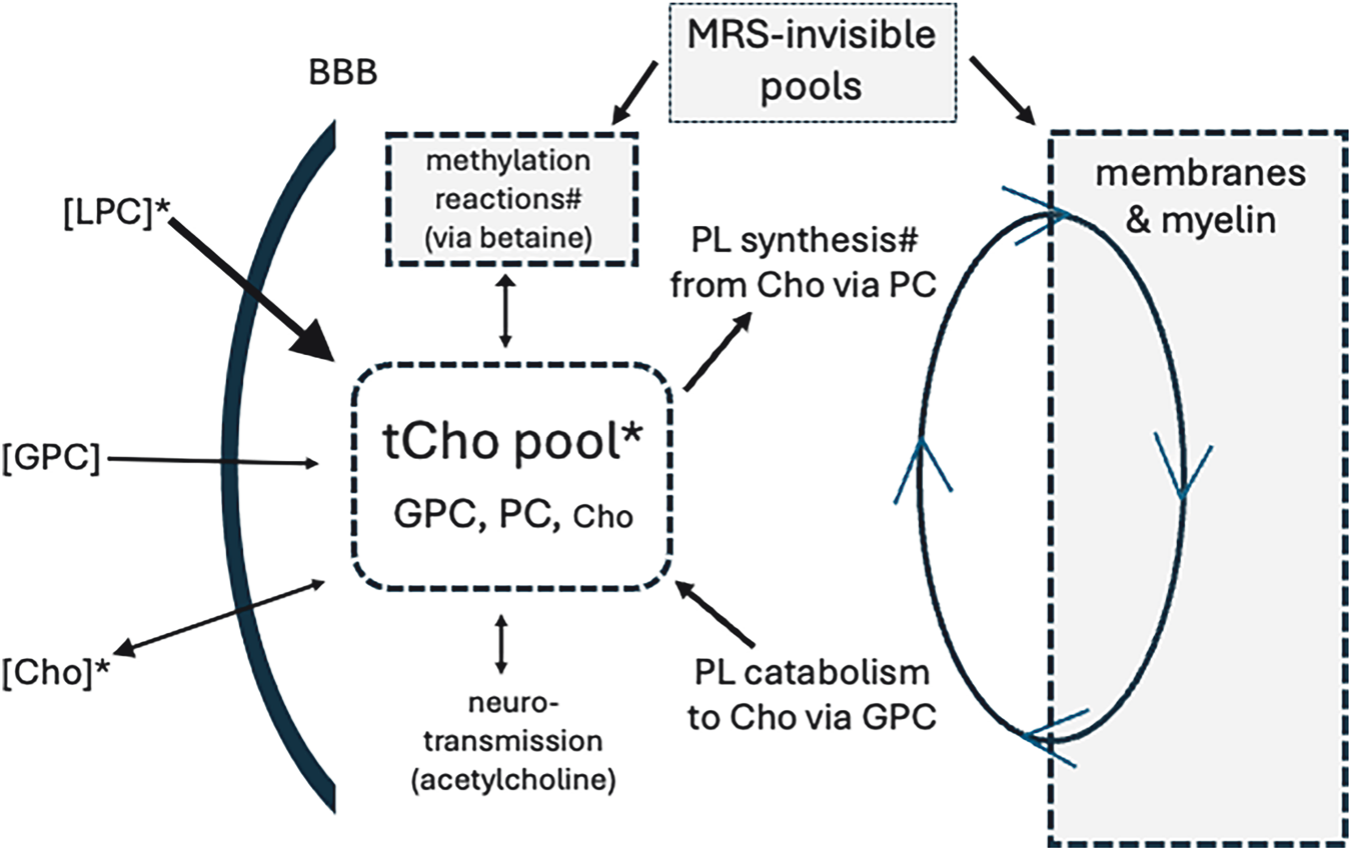
Overview of factors influencing MRS-visible tCho signal. * Indicates reduced levels have been associated with anxiety. # Indicates where choline moieties moving into MRS-invisible pools has been associated with noradrenergic activity. [Brackets] indicate compounds in systemic circulation. BBB blood-brain barrier, LPC lysophosphatidylcholine (primary form of choline crossing BBB), GPC glycerophosphocholine, PC phosphocholine, Cho free choline (net efflux from brain), tCho MRS-visible choline-containing compounds (mostly GPC and PC), PL choline-containing phospholipids (in ongoing cycle of synthesis, remodeling, and breakdown with PC and GPC).

Three general processes influence brain tCho levels in the absence of overt brain injury, neoplasm or degeneration. These include changes in the dynamic cycling of choline-containing phospholipids in membranes and myelin [69], changes in the uptake of choline moieties into the brain and efflux out of the brain [70, 71], and changes in the consumption or synthesis of choline moieties within the brain as part of methylation reactions [72, 73] (Fig. 4). Acetylcholine levels also have a minor contribution to tCho levels. Reduced brain tCho levels in AnxDs could be associated with one or more of these processes.

### Dynamic cycling of membrane phospholipids

Glycerophosphocholine (GPC), phosphocholine (PC), and (to a lesser extent) free choline are the primary sources of the tCho signal [13]. All are soluble intermediates in the cycle of synthesis and breakdown of choline-containing membrane phospholipids, including phosphatidylcholine (PTC) and sphingomyelin. PTC is the most abundant of these and contains most of the choline moieties in the brain [74]. Unlike GPC, PC, and free choline, the choline moieties in PTC and sphingomyelin are confined to membranes and thus not visible to MRS [13]. Intracellular trafficking and remodeling of membrane phospholipids is an indispensable neurobiological process [69]. Since this requires essentially continuous synthesis and breakdown of PTC, the compounds giving rise to the tCho signal are best understood as part of a dynamic metabolic cycle that includes the membrane-bound choline moieties [69].

### Brain uptake and efflux of choline moieties

Among the metabolite moieties readily measured by 1H-MRS in clinical studies, only the choline moiety is minimally synthesized within the brain and thus dependent on uptake from the systemic circulation [70]. Although the liver synthesizes some choline, dietary intake is the main source of systemic choline compounds [75]. Interestingly, 90% of adults in the US fail to consume the recommended adequate daily amount of choline [76], and lower levels of serum choline were associated with clinically significant anxiety in a study of almost 6000 subjects [77]. A blood-brain-barrier (BBB) transporter for lyso-phosphatidylcholine (LPC) has been identified, which appears to be the major route for systemic choline moieties entering the brain [71, 78, 79]. Once in the brain, the choline in LPC contributes to the tCho signal by entering the membrane phospholipid cycle and to a lesser extent by hydrolysis to choline and acetylation to acetylcholine [13, 71, 78]. It is worth noting that low serum levels of LPC predict more anxious behavior in a rat model of diet-induced anxiety [80]. A BBB transporter for free choline has also been identified, which may function primarily to export choline from the brain [81]. This view is consistent with arterio-venous difference studies showing that the brain is a net exporter of free choline, with the rate of choline efflux varying depending on metabolic conditions [70, 79].

### Methylation reactions

Methylation reactions via s-adenosylmethionine (SAM), including epigenetic modifications via DNA and histone methylation, are also related to brain levels of choline compounds. Choline is an important methyl donor for SAM [72]. McCoy et al. [82] found reduced DNA methylation in the amygdala in an anxious strain of rats and showed that dietary supplementation or depletion with choline and other methyl donors led to decreased or increased anxiety, respectively. Methyltransferase reactions via SAM also synthesize some choline de novo in the brain [73].

### Possible arousal-related effects on tCho in anxiety disorders

Chronically elevated arousal is a hallmark of AnxDs. Elevated activity of arousal-related neuromodulating systems, including noradrenergic, cholinergic and orexinergic systems, has been implicated in these disorders and triggers neurometabolic changes [83–92]. The metabolic consequences of elevated noradrenergic activity serve as one example of this possible mechanism, as they directly influence adult brain choline-related metabolism in several ways. Noradrenergic neuromodulation promotes activation, proliferation, and differentiation of cortical oligodendrocyte precursor cells in support of myelination [93, 94], similar to the effects of thyroid hormone. If the increased demand for choline moieties for myelin synthesis is not accompanied by a proportionate increase in uptake of choline moieties across the BBB, brain tCho signal could be reduced. In neurons, noradrenergic activity stimulates methylation reactions that consume methyl groups from SAM without leading to the synthesis of choline moieties [95, 96]. Since choline is a methyl donor for SAM, this change could decrease the tCho signal. In astrocytes, noradrenergic activity shifts glucose metabolism toward aerobic glycolysis and increased lactate production [97], a phenomenon that has been demonstrated in panic disorder patients [30, 36]. While not a direct factor in choline metabolism, this observation is consistent with heightened noradrenergic activity having neurometabolic effects in an anxiety disorder. Empirically, reduced brain tCho has been reported in some conditions associated with elevated noradrenergic activity. There is a well-established synergism between metabolic effects of noradrenaline and those of thyroid hormone (TH) [98, 99]. Studies of hyperthyroid patients demonstrate reduced tCho across several brain regions with robust effect sizes [16, 100–102]. Conversely, hypothyroid patients show consistently elevated tCho [103–105]. In addition to its essential role in neurodevelopment, TH regulates many neurometabolic pathways in adults [106], including such choline-related processes as myelin turnover, maturation of oligodendrocyte precursor cells, and degradation of membrane phospholipids (via regulation of phospholipases) [107–110]. Similarly, vigorous physical activity strongly evokes increased central noradrenergic neuromodulation, and higher levels of regular physical activity are associated with lower brain tCho levels [111–115], an effect that may be specific to GPC levels [116].

The above considerations illustrate the possibility that reduced tCho in AnxDs may develop, in part, as a metabolic consequence of chronically elevated arousal-related neuromodulation. If so, an intriguing possibility is that arousal-induced changes in the metabolic disposition of brain choline moieties leads to increased choline entry into MRS-invisible compartments without a proportionate increase in soluble choline uptake across the BBB. In this regard, lower serum levels of choline compounds have been associated with anxiety [77, 80]. Both hypothesized factors, elevated arousal-related neuromodulation and a relative shortfall of brain uptake, would predict a widespread effect on tCho levels, which corresponds to the reduced tCho seen generally across cortical regions in AnxDs. Widespread or trans-regional metabolic influences may be a characteristic feature of brain tCho levels. Rodríguez-Nieto et al. [117] recently reported on the degree of inter-regional homogeneity of NAA, tCr, tCho, myo-inositol, and glx levels across seven brain regions. They found that only tCho levels were strongly correlated across the seven regions. Their metric for degree of metabolic connectivity was 5.8 and 8.1 times higher for tCho than for any other metabolite in young and older individuals, respectively. Dietary intake of choline falls short of recommended adequate levels for most adults [76]. If brain choline uptake fails to satisfy increased metabolic demand in AnxDs, it is possible that the resulting brain choline shortfall itself becomes a pathogenic factor that promotes anxiety or impedes recovery. If so, then appropriate choline supplementation could have therapeutic value for people with these disorders. Although clinical benefit from choline supplementation for AnxDs has not been tested in controlled trials, indirect evidence supports the possibility. Models of recovery from AnxDs invoke new learning at the cortical level [118, 119]. Animal studies show that choline deprivation impedes while supplementation improves neuroplasticity and learning [120, 121], and clinical studies of aging and memory-impaired populations consistently show improved memory associated with greater dietary intake of choline or choline supplementation [122–125]. Choline enters the brain primarily in the form of LPCs containing an omega-3 fatty acid (O3FA) [71, 78, 79]. Extensive research shows that O3FA supplementation improves anxiety symptoms, most clearly in people with anxiety disorders [126, 127]. Since O3FAs enter the brain primarily in the form of LPCs (which also contain choline) [128], augmenting brain O3FA uptake simultaneously increases brain choline uptake. Controlled trials combining 1H-MRS with choline supplements, perhaps as LPCs containing O3FAs, could formally test the hypothesis that correcting low brain tCho in AnxDs is associated with therapeutic benefit.

### NAA findings

The current meta-analysis found NAA in the prefrontal cortex did not differ between AnxD patients and controls, disconfirming the prediction that low prefrontal NAA would implicate executive dysfunction as a transdiagnostic feature of AnxDs. After excluding outlier datasets, a small but significant reduction in NAA was seen across all cortical regions, suggesting some degree of compromised neuronal function in people with AnxDs. Larger and more consistent reductions in cortical NAA are seen in schizophrenia [21], along with clear histological evidence of anti-trophic changes in cortical neurons and astrocytes and imaging evidence of cortical thinning [129–131]. In contrast, recent multi-center mega-analyses found little evidence of cortical thinning in SAD or GAD [132, 133]. Further studies would be necessary to clarify the consistency, extent, and potential significance of reduced cortical NAA in AnxDs.

### Creatine findings

An exploratory analysis of nine datasets showed a significant effect of medication status on cortical tCr values, with tCr significantly reduced in the five studies of unmedicated patients. Importantly, only one of the nine studies reporting cortical tCr corrected the water signal for CSF partial volume, which is a minimum requirement for appropriate water-normalization of metabolites [134]. In spite of limitations to the generalizability of the current creatine finding, it suggests that investigators reporting creatine-normalized metabolites in AnxD studies also report appropriately water-normalized values or demonstrate equivalence of water-normalized creatine values in patients and controls.

### Measurement quality, study quality and heterogeneity

Quality of the metabolite measurements (indexed by COV), significantly moderated effect sizes for cortical tCho but not NAA, with greater reductions in tCho datasets with lower COVs. COV is an indirect measure of metabolite measurement quality. It was the only quality metric available for analysis, as very few studies reported mean values for direct metrics such as linewidth or CRLB. Mean tCho COV in the empirically-identified datasets with better measurement quality was 13%, with a 95% confidence interval extending up to 17%. A recent meta-analysis demonstrating elevated tCho in schizophrenia found a similar moderating effect of COV on effect sizes [21]. This study also reported a moderating effect of CRLB, such that datasets reporting a mean CRLB ≤ 3 for tCho observed significantly greater effect sizes. Future studies of the clinical and pathophysiological significance of tCho will benefit from stricter linewidth and CRLB inclusion thresholds than those in common use and stricter exclusion rules for distorted spectra and outlier values. Similarly, our rating of overall study quality significantly moderated cortical tCho effect sizes, which were greater in studies with higher overall quality. Significant heterogeneity observed in cortical tCho and NAA meta-analyses could not be attributed to any of the moderating factors we examined, but appeared to result from statistical outliers. Heterogeneity was low and non-significant when these were excluded.

### Limitations

Our analysis had several limitations. First, the relatively small number of studies reporting on metabolites other than NAA and tCho diminished our power to detect consistent regional abnormalities in those metabolites, especially GABA. Similarly, very few studies investigated subcortical threat processing regions. The limited number of datasets particularly constrained our ability to examine potentially moderating factors, including differences between specific diagnoses, medication status, and data acquisition and processing methods. Only when combing data across all cortical regions was there adequate power to examine such factors. Some important variables, such as age, symptom severity, comorbidity, and medication dosage are difficult to examine in aggregated data by meta-analysis and are best approached using individual patient data. In addition, most studies did not report standard measurement quality metrics (e.g. line width and CRLB). Only COV, an indirect quality metric, was available for moderator analyses. Finally, a critical, unanswered question is whether reduced cortical tCho is associated with clinical severity or prognosis in AnxDs, and if so, if the direction of the association suggests a pathogenic or a beneficially adaptive process. Patient-level data will be required to address this question.

## Conclusions

This first ever meta-analysis of 1H-MRS studies of people with anxiety disorders (SAD, GAD, and PD) identified a transdiagnostic pattern of reduced choline-containing compounds in the prefrontal cortex and across cortical regions generally. The effect size was moderated by metabolite measurement quality, with cortical tCho levels in patients averaging 8% lower than in controls across datasets with tCho COV ≤ 17%. The effects of arousal-related neuromodulators, such as norepinephrine, on choline metabolism in neurons and glial cells suggest a possible mechanism for reduced cortical tCho in AnxDs. Further studies will be required to clarify the mechanism and clinical significance of this transdiagnostic abnormality, including the possibility of therapeutic benefit from appropriate choline supplementation.

## Supplementary information

Supplemental Materials
