# Supplementary material for: Transdiagnostic reduction in cortical choline-containing compounds in anxiety disorders: a 1H-magnetic resonance spectroscopy meta-analysis
Source: Mol Psychiatry. 2025 Sep 5;30(12):6020–32. doi: 10.1038/s41380-025-03206-7 (PMC12602319; doi:10.1038/s41380-025-03206-7)
Supplement: Supplementary file 1 — Supplemental Materials [file 41380_2025_3206_MOESM1_ESM.docx]

Supplemental Materials for:

"Transdiagnostic Reduction in Cortical Choline-Containing Compounds in Anxiety Disorders: A 1H-Magnetic Resonance Spectroscopy Meta-Analysis"

Richard J. Maddock, MD and Jason Smucny, PhD

Page 1: Table of Contents

Page 2: Figure S1 - PRISMA flow diagram

Pages 3-4: Supplemental Methods

Pages 5-7: Table S1 - MRS methods for included and excluded studies

Pages 8-9: Table S2 - Demographic and clinical features of included studies

Page 10: Table S3 - Study quality checklist for MRS studies of Anxiety Disorders

Pages 11-12: Table S4 - Prevalence of psychiatric comorbidities

Page 13: Table S5 Moderation of cortical metabolite effect sizes by diagnosis

Page 14: Table S6 - Moderation of cortical metabolite effect sizes by medication status

Pages 15-16: Table S7 - Moderation of effect sizes by normalization, % male, age, echo time & study quality

Page 17: Figure S2 - Forest plots for tCho from additional brain regions

Pages 18-20: Figure S3 - Forest plots for NAA from additional brain regions

Pages 21-22 Figure S4 - Forest plots for total creatine

Pages 23-24 Figure S5 - Forest plots for myo-inositol

Page 25 Figure S6 - Forest plots for glutamate

Page 26 Figure S7 - Forest plots for Glx

Page 27 Figure S7 - Forest plots for GABA and lactate

Pages 28-31 Supplemental Results

Pages 32-35 References for Supplemental Materials

**
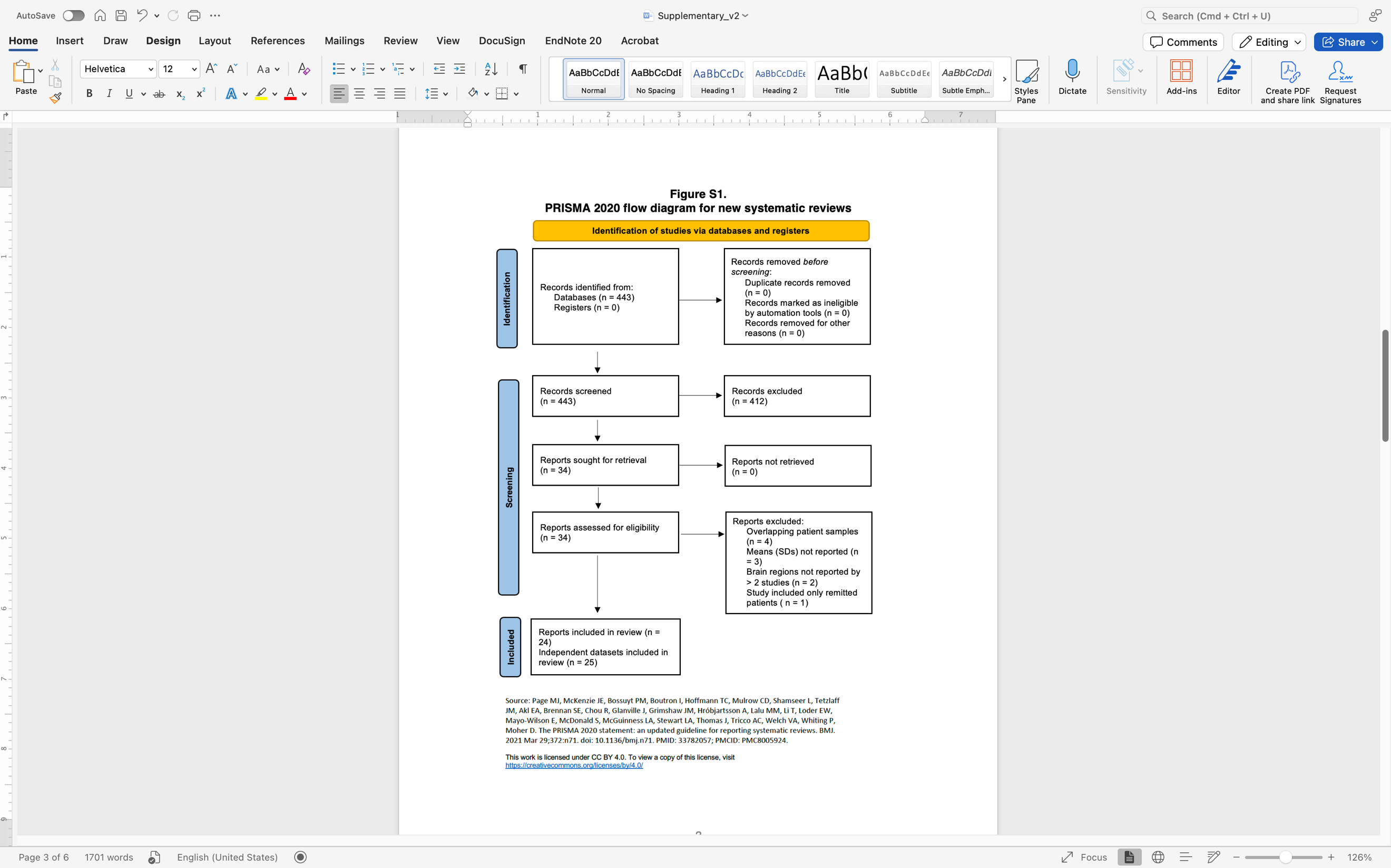
**

**Supplemental Methods**

*Empirical method for testing the moderating effect of metabolite measurement quality in 1H-MRS meta-analyses.*

As observed in prior 1H-MRS meta-analyses (1,2), we anticipated that true group differences in regional metabolite values would be most evident in studies with relatively better metabolite measurement quality. That is, pooled effect sizes would become larger and more consistent above an identifiable measurement quality threshold. We considered four metrics to be sensitive to the quality of metabolite measurements: coefficient of variation (COV, standard deviation/mean) of metabolite values, Cramer-Rao lower bound (CRLB) for metabolite fits, singlet line width (FWHM), and singlet signal-to-noise ratio (SNR). Only the COV metric was available for ≥ 14 datasets, which is customarily the minimum for this moderator analysis (1,2). For COV, we averaged the COV values for the patient and control groups in each dataset. COV is an indirect metric for the precision of metabolite measurements. It reflects the variance due to true differences across subjects combined with the variance due to measurement noise from all sources. Thus, lower COVs generally reflect less measurement noise and better measurement quality. We hypothesized that measurement quality would have a moderating effect on the meta-analytic results across studies comparing anxiety disorder patients to healthy volunteers, and that the relationship between COV and effect size would be logistic (sigmoid), rather than linear. To test this hypothesis, we first ranked the studies from lowest to highest quality based on COV. We then calculated the inverse variance-weighted pooled effect sizes (as Hedge’s *g*) from successive subsamples of k' datasets, such that each successive *g* value was calculated from a subsample of datasets with slightly better measurement quality than the preceding subsample. The value of k' = total k/5, or k' = 7, whichever is greater. For the current analysis of cortical tCho and cortical NAA, k' = 7. The successive meta-analyses of subsamples ran from the k’ lowest quality studies to the k’ highest quality studies (analogous to a moving average). A best-fitting, 4-parameter, logistic function was fit to this series of pooled effect sizes using the computational resource at <https://findcurves.com/> using the following equation:

Y = d + ((a – d)/(1+(X/c)^b))

Where Y = the pooled effect size (of k’ studies) and X = the rank of the set of k’ adjacent studies for the quality metric (COV). Parameter “a” is the asymptote of the pooled effect size (*g*) for the lowest quality datasets, and parameter “d” is the asymptote of the pooled effect size (*g*) for the highest quality datasets for each metric. These best fitting parameters were used to generate a logistic transform of the ranks of the k’ adjacent studies based on COV.

The inflection point (IP) is the value of *g* at the midpoint between parameters “a” and “d” (thus IP = (a + d)/2). This point IP was used to stratify studies into low and high quality subgroups for each metric. The mean and standard deviation of the COV were calculated for all datasets contributing to effect sizes *g* (pooled across k' datasets) that were stronger than the inflection point *g*. The threshold (T) for identifying an individual dataset as "better measurement quality) was defined as

COV ≤ X̅ + Z*(SD/√k)

where X̅ and SD = mean and standard deviation of the COV for all datasets contributing to effect sizes *g*  that were stronger than the inflection point *g;* Z = 1.645, which is the one-tailed Z value for a 95% confidence interval; k = number of datasets contributing the mean and SD.

The best fitting four parameters (a, b, c, and d) for COV and results of the regression against the logistic curve are shown in the Table below for cortical tCho, which was the only regional metabolite demonstrating significant moderating effects of measurement quality indexed by COV.

|  |  | a | b | c | d | *r ^2^* | *p* | IP (*g*) | T | x̅ low COV | x̅ high COV |
| --- | --- | --- | --- | --- | --- | --- | --- | --- | --- | --- | --- |
| **Cortical tCHo** | k |  |  |  |  |  |  |  |  |  |  |
| COV all datasets | 16 | -.229 | 174 | 6.02 | -.382 | .61 | .035 | -.31 | 18% | 12.6% (10) | 21.9% (6) |
| COV excl outliers | 14 | -.302 | 6.39 | 5.76 | -.660 | .96 | >.001 | -.48 | 18% | 13.0% (8) | 21.9% (6) |

*r ^2^* = the square of the adjusted coefficient of regression, *p* = significance level, IP = the logistic inflection point in units of Hedges' *g*, T = COV quality threshold, as defined above; x̅ low COV = mean COV value for studies with COV ≤ T (number of datasets), x̅ high COV = mean COV value for studies with COV > T (number of datasets).

**Table S1.** Magnetic resonance spectroscopy (MRS) methods for datasets and brain regions included in analyses. Abbreviations: B = bilateral, Cho = phosphocholine+glycerophosphocholine, Cr = creatine+phosphocreatine, DLPFC = dorsolateral prefrontal cortex, DMPFC = dorsomedial prefrontal cortex, Gln = glutamine, Glx = glutamate+glutamine, GM = gray matter, L = left, M = medial, mPFC = medial prefrontal cortex, Myo = myo-inositol, NAA = n-acetyl-aspartate, NR = not reported, OFC = orbitofrontal cortex, PFC = prefrontal cortex, R = right, VMPFC = ventromedial prefrontal cortex, WM = white matter.

| **Study** | **Regions(s) Reported** | **Metabolite(s) Reported** | **Normalization** | **Lateraliza-tion for Each Region** | **Acquisition (Method, Field Strength, TE, TR)** | **Single or Multi-Voxel** | **Excluded?**  **(reason)** |
| --- | --- | --- | --- | --- | --- | --- | --- |
| ***GAD*** | | | | | | |  |
| Mathew et al. (2004) (3) | DLPFC | Cho, NAA | Cr | B | PRESS, 1.5T, TE/TR = 280/2300 | Multi |  |
| Mathew et al. (2008) (4) | Hippocampus | Cr, NAA | Phantom Replacement | B | PRESS, 1.5T, TE/TR = 280/2300 | Multi |  |
| Coplan et al. (2006) (5) | Cortical WM | Cr, NAA, Cho | Phantom Replacement | B | PRESS, 1.5T, TE/TR = 280/2300 | Multi |  |
| Coplan et al. (2018) (6) | Hippocampus, | Cho | Cr | B | PRESS, 3T, TE/TR = 280/2300 | Multi |  |
| Hettema et al. (2012) (7) | Amygdala, Hippocampus, mPFC, OFC | Glx, Cr, NAA, Cho, Myo | Water | B, B, M, L | PRESS, 3T, TE/TR = 35/1500 | Single |  |
| Strawn et al. (2013) (8) | mPFC | Glu | Cr, Water | M | PRESS, 4T, TE/TR = 280/3000 | Single |  |
| Moon et al. (2015) (9) | DLPFC | Cho, Glx, Lactate, Myo, NAA | Cr | R | PRESS, 3T, TE/TR = 30/2000 | Single | Excluded Lactate only |
| Raparia et al. (2016) (10) | Premotor Cortex, Rostral PFC, Sensorimotor Cortex | Cho, Cr, NAA | Phantom Replacement | B, B, B | PRESS, 3T, TE/TR = 280/2300 | Multi |  |
| Pigoni et al. (2020) (11) | DLPFC | Cho, Cr Glu, Myo, NAA | Water | R | PRESS, 1.5T, TE/TR = 80/4000 | Single |  |
| Mathew et al. (2009) (12) | Lateral Ventricles | Lactate | Root-Mean-Square Noise | B | PRESS, 3T, TE/TR = 280/2300 | Multi | Excluded^3^ |
| Abdallah et al. (2012) (13) | Occipital | NAA | Phantom Replacement | B | PRESS, 1.5T, TE/TR = 280/2300 | Multi | Excluded^2^ |
| Abdallah et al. (2013) (14) | Hippocampus | NAA | Phantom Replacement | B | PRESS, 1.5T, TE/TR = 280/2300 | Multi | Excluded^1^ |
| Coplan et al. (2014) (15) | Hippocampus | NAA | Phantom Replacement | B | PRESS, 1.5T & 3T, TE/TR = 280/2300 | Multi | Excluded^1,2^ |
| Moon and Jeong (2016) (16) | DLPFC | Cho, Glx, Lactate, Myo, NAA | Cr | R | PRESS, 3T, TE = 30 ms, TR = 2000 ms | Single | Excluded^1^ |
| Moon et al. (2016) (17) | DLPFC | Cho, Glx, Lactate, Myo, NAA | Cr | R | PRESS, 3T, TE = 30 ms, TR = 2000 ms | Single | Excluded^1^ |
| ***PD*** | | | | | | |  |
| Dager et al. (1997) (18) | Insula | Lactate | NAA | L | PRESS, 1.5T, TE/TR = 272/2000 | Single |  |
| Goddard et al. (2001) (19) | Occipital | GABA | Cr | M | MEGAPRESS, 2.1T, TE/TR = 68/3390 | Single |  |
| Massana et al. (2002) (20) | Medial Temporal, mPFC | Cho, Cr, NAA | Cr, Water | R, M | PRESS, 1.5T, TE = 25-35 ms, TR = 1500 ms | Single |  |
| Ham et al. (2007) (21) | ACC, Basal Ganglia | Cho, Cr Glx, Myo, NAA | Water | M, L | PRESS, 3T, TE/TR = 35/2000 | Single | Excluded GABA & Lactate only |
| Hasler et al. (2009) (22) | DMPFC, VMPFC | Cho, GABA, Glx, NAA | Cr | B, B | PRESS, 3T, TE/TR = 68/1500 | Single |  |
| Maddock et al. (2009) (23) | Occipital | Cr, Lactate, NAA, Cho | Cr, Water | M | PRESS, 1.5T, TE/TR = 288/1500 | Single |  |
| Maddock et al. (2013) (24) | Occipital | Cr, Glx, Cho, Lactate, NAA | Cr, NAA | M | BASING-PRESS, 1.5T, TE/TR = 144/1500 | Single | Excluded data from remitted pts |
| Trzesniak et al. (2010) (25) | Hippocampus | Cho, NAA | Cr | L, R | PRESS, 1.5T, TE/TR = 270/1500 | Multi |  |
| Long et al. (2013) (26) | mPFC, Occipital | GABA | Cr | M, M | PRESS, 3T, TE/TR = 68/1500 | Single |  |
| Pigoni et al. (2020) (11) | DLPFC | Cho, Cr Glu, Myo, NAA | Water | R | PRESS, 1.5T, TE/TR = 80/4000 | Single |  |
| Dager et al. (1994) (27) | Insula | Lactate | NAA | L | PRESS, 1.5T, TE/TR = 272/2000 | single | Excluded^2^ |
| Dager et al. (1995) (28) | Insula | Lactate | NAA | L | PRESS, 1.5T, TE/TR = 272/2000 | Single | Excluded^4^ |
| Dager et al. (1999) (29) | Multiple regions | Lactate | NAA | L | PEPSI, 1.5T, TE/TR = 272/2000 | Multi | Excluded^2^ |
| ***SAD*** | | | | | | |  |
| Davidson et al. (1993) (30) | Caudate, Cortical WM | NAA | Cr | NR | STEAM, 1.5T, TE/TR = 270/2000 | Multi |  |
| Tupler et al. (1997) (31) | Cortical GM, Cortical WM, Subcortical | Cho, Myo, NAA | Cho, Cr | B, B, B | STEAM, 1.5T, TE/TR = 20/1500 | Multi |  |
| Phan et al. (2005) (32) | mPFC, Occipital | Cho, Glu, NAA | Cr | M, M | STEAM, 4T, TE/TR = 10/2000 | Single |  |
| Yue et al. (2012) (33) | DLPFC, mPFC, Putamen, Thalamus | Cho, Cr, NAA | Cr, Water | L, M, B, L | STEAM, 3T, TE/TR = 20/2000 | Single |  |
| Howells et al. (2015) (34) | mPFC | Cho, Glu, Glx, Myo, NAA | Cr | M | PRESS, 3T, TE/TR = 30/1500 | Single |  |
| Also Howells et al. (2015), multivoxel acquisition (34) | Caudate, Putamen, Thalamus | Cho, NAA | Cr | B, B, B | PRESS, 3T, TE/TR = 30/2000 | Multi |  |
| Tukel et al. (2016) (35) | Caudate, Insula, mPFC, Putamen | Cho, Myo, NAA | Cr | L, L, L, L | STEAM, 1.5T, TE/TR = 30/2500 | Single |  |
| Pollack et al. (2008) (36) | Thalamus, Whole Brain | GABA, Gln, Glu | Cr | M, B | J-RES, 4T, TE = 30-490, TR = 1400 | Multi | Excluded^3^ |

^1^ Excluded from meta-analysis due to extensive overlap of subject sample with a larger, included study

^2^ Excluded from meta-analysis because means and SDs of metabolite levels were not reported

^3^ Excluded from meta-analysis because reported on regional metabolites on which ≤ 2 other studies reported

^4^Excluded from meta-analysis because included only remitted patients

**Table S2.** Demographic and clinical characteristics of included studies (Hamilton Anxiety Scale (HAM-A) mean and standard deviation (SD) scores and %medicated with antidepressants at time of scan). Numbers without parentheses represent the mean unless otherwise specified. Numbers in parentheses represent the SD. Information does not consider participants who were removed from individual studies due to data quality or other factors. Abbreviations: %M = percent male, AnxD = anxiety disorder, GAD = generalized anxiety disorder, HC = healthy control(s); NR = not reported, PD = panic disorder, SAD = social anxiety disorder, Std Q = Study Quality rating (range = 0 - 14).

| **Study** | **HC** | | | **AnxD** | | | | |  |
| --- | --- | --- | --- | --- | --- | --- | --- | --- | --- |
|  | ***n*** | ***Age in Years*** | ***%M*** | ***n*** | ***Age in Years*** | ***%M*** | **HAM-A** | **%Medicated** | **Std Q** |
| ***GAD*** | | | | | | | | |  |
| Coplan et al. (2006) (5) | 15 | 39.1 (13.5) | 47 | 15 | 39.3 (13.6) | 47 | 22.6 (3.5) | 0 | 11 |
| Coplan et al. (2018) (6) | 16 | 36.5 (11.1) | 38 | 16 | 38.1 (13.2) | 31 | NR | 0 | 10 |
| Hettema et al. (2012) (7) | 17 | Median = 52 | 0 | 17 | Median = 45 | 0 | NR | 59 | 9 |
| Mathew et al. (2004) (3) | 15 | 39.1 (13.5) | 47 | 15 | 39.3 (13.3) | 47 | 22.6 (3.5) | 0 | 11 |
| Mathew et al. (2008) (4) | 8 | 27.4 (4.2) | 38 | 15 | 31.7 (9.6) | 40 | 20.0 (3.6) | 0 | 11 |
| Moon et al. (2015) (9) | 15 | 38.8 (8.9) | 60 | 15 | 35.4 (9.6) | 60 | 17.8 (5.2) | 93 | 12 |
| Pigoni et al. (2020) (11) | 16 | *n =* 9 ≤ 30  3 > 30 and ≤ 45  4 > 45 | 25 | 10 | *n =* 3 ≤ 30  4 > 30 and ≤ 45  3 > 45 | 30 | 20.4 (10.5) | use of medications not an exclusion | 9 |
| Raparia et al. (2016) (10) | 16 | 36.5 (11.1) | 38 | 16 | 38.1 (13.3) | 31 | NR | 0 | 10 |
| Strawn et al. (2013) (8) | 10 | 13 (3) | 40 | 10 | 14 (2) | 40 | 24 (6) | 0 | 10 |
| ***PD*** | | | | | | | | |  |
| Dager et al. (1997) (18) | 13 | 37.7 (7.5) | 40 | 10 | 36.2 (9.1) | 38 | NR | 0 | 11 |
| Goddard et al. (2001) (19) | 14 | NR | 43 | 14 | 37 (10) | 43 | 17 (8) | 0 | 10 |
| Massana et al. (2002) (20) | 11 | 34.6 (7.0) | 45 | 11 | 34.3 (6.9) | 45 | NR | 0 | 10 |
| Ham et al. (2007) (21) | 25 | 30.5 (5.2) | 58 | 22 | 31.9 (6.8) | 58 | NR | 100 | 9 |
| Hasler et al. (2009) (22) | 17 | 35.1 (11.8) | 29 | 17 | 34.2 (10.1) | 29 | 9.6 (6.2) | 0 | 13 |
| Maddock et al. (2009) (23) | 15 | 37.1 (6.9) | 33 | 15 | 37.5 (9.2) | 33 | NR | 0 | 12 |
| Maddock et al. (2013) (24) | 12 | 38.4 (8.7) | 25 | 21 | 38.7 (11.3) | 33 | NR | 0 | 14 |
| Trzesniak et al. (2010) (25) | 18 | 35.7 (12.5) | 33 | 25 | 39.2 (9.9) | 24 | NR | 52 | 11 |
| Long et al. (2013) (26) | 8 | 39.5 (12.1) | 50 | 11 | 38.2 (12.7) | 55 | NR | 0 | 9 |
| Pigoni et al. (2020) (11) | 16 | *n =* 9 ≤ 30  3 > 30 and ≤ 45  4 > 45 | 25 | 11 | *n =* 5 ≤ 30  3 > 30 and ≤ 45  3 > 45 | 27 | 14.3 (7.0) | use of medications not an exclusion | 10 |
| ***SAD*** | | | | | | | | |  |
| Davidson et al. (1993) (30) | 20 | 34.6 (9.1) | 50 | 20 | 35.7 (6.7) | 55 | NR | 0 | 11 |
| Tupler et al. (1997) (31) | 10 | 37.8 (10.5) | 60 | 19 | 42.0 (11.6) | 26 | NR | 0 | 10 |
| Phan et al. (2005) (32) | 10 | 26.6 (6.8) | 50 | 10 | 26.7 (6.8) | 50 | NR | 0 | 13 |
| Yue et al. (2012) (33) | 9 | 21.2 (2.0) | 56 | 9 | 21.6 (2.5) | 56 | NR | 0 | 10 |
| Howells et al. (2015) (34) | 19 | 29.2 (8.2) | 58 | 18 | 31.0 (9.9) | 39 | NR | 0 | 11 |
| Tukel et al. (2016) (35) | 24 | 28.4 (5.8) | 50 | 24 | 28.5 (6.63) | 50 | 6.9 (5.5) | 0 | 12 |

**Table S3**: Study quality checklist for MRS studies of Anxiety Disorders

Author_________________ year_________

| **Category 1: Sample characteristics and matching (7)** | **score** |
| --- | --- |
| 1. Patients were evaluated with appropriate standardized diagnostic criteria (1) |  |
| 1. Healthy comparison subjects were evaluated to exclude psychiatric and medical illnesses (1) |  |
| 1. Age and sex are reported with mean (or median) and standard deviations (or range) and these are comparable in the anxiety disorder and healthy comparison participants (1) |  |
| 1. Whether patients currently meet diagnostic criteria is reported in sufficient detail (1) |  |
| 1. Medication status of patients is reported in sufficient detail (1) |  |
| 1. Comorbidity disorders in patients are reported in sufficient detail (1) |  |
| 1. Total sample size for patients and controls is > 20 and neither group is < 8 (1) |  |
| **Subtotal (out of 7)** |  |
| **Category 2: MRS Methodology and reporting (7)** |  |
| 1. Volume of interest (VOI) locations are reported in sufficient detail (1) |  |
| 1. Magnet strength at least 1.5T for most metabolites (if less than 3T for reporting glutamate, then exclude glutamate data) (1) |  |
| 1. Pulse sequence and basic scan parameters are reported, including TE, TR, & NEX, for conventional scans and editing frequencies and edit pulse bandwidth for edited scans. If GABA, lactate, or GSH are reported without use of an optimized MRS acquisition protocol, such data are excluded. (1) |  |
| 1. MRS processing techniques and quantification methods are clearly described (1) |  |
| 1. Grey vs. white matter proportions in the MRS voxel are reported and comparable between groups or are quantitatively incorporated into the statistical model for comparing metabolites between groups (1) |  |
| 1. Metabolite referencing to water includes a correction for CSF fraction in the voxel, or creatine referencing is used. Metabolite data referenced using other than creatine, NAA, water, or phantom replacement are excluded. (1) |  |
| 1. Measurement quality metrics for the spectra or the metabolites (either FWHM of NAA or Cr, or metabolite CRLBs) are reported quantitatively and comparable between groups. (1) |  |
| **Subtotal (out of 7)** |  |
| **Total score (14)** |  |

**Table S4**. Prevalence of psychiatric comorbidities in each study.

CC: Comorbidity classification (0 = none; 1 = minor; 2 = substantial, > 25% of sample; NR = comorbidity data not reported

| **Study** | ***n* in Anxiety Group** | **Current Comorbidities in Anxiety Group** | **CC** |
| --- | --- | --- | --- |
| ***GAD*** | | |  |
| Coplan et al. (2006) (5) | 15 | Dysthymia (*n=*5), Social Anxiety Disorder (*n*=2), Social Anxiety Disorder with Dysthymia (*n*=1) | 1 |
| Coplan et al. (2018) (6) | 16 | Current secondary Major Depression allowed, no other information about comorbid conditions | 2 |
| Hettema et al. (2012) (7) | 17 | Major Depression (*n*=11), Phobias (*n*=3), Panic Disorder (*n*=1), all as lifetime Dx's, no information on current Dx's | NR |
| Mathew et al. (2004) (3) | 15 | Dysthymia (*n=*5), Social Anxiety Disorder (*n*=2), Social Anxiety Disorder with Dysthymia (*n*=1) | 1 |
| Mathew et al. (2008) (4) | 14 | Panic Disorder (*n*=6), Dysthymia (*n*=5), Social Anxiety Disorder (*n*=3) | 2 |
| Moon et al. (2015) (9) | 15 | Mild Depression (*n*=15), no other information about comorbid conditions | NR |
| Pigoni et al. (2020) (11) | 10 | Confirms the absence of any comorbid conditions | 0 |
| Raparia et al. (2016) (10) | 14 | Current secondary Major Depression allowed, no other information about comorbid conditions | 2 |
| Strawn et al. (2013) (8) | 10 | No information about comorbid conditions (exclusion criteria: substance dependence, posttraumatic stress disorder, bipolar disorder, psychosis, obsessive-compulsive disorder, pervasive developmental disorder, neurological disorders) | NR |
| ***PD*** | | |  |
| Dager et al. (1997) (18) | 13 | Confirms the absence of any comorbid conditions | 0 |
| Goddard et al. (2001) (19) | 14 | Somatoform disorder (1), social phobia, specific subtype (1) | 1 |
| Massana et al. (2002) (20) | 11 | Confirms the absence of any comorbid conditions | 0 |
| Ham et al. (2007) (21) | 22 | Confirms the absence of any comorbid conditions "requiring psychotropic medications" or substance abuse | 0 |
| Hasler et al. (2009) (22) | 17 | Major Depressive Disorder (*n*=7), Agoraphobia (*n*=7), Phobic Disorder (*n*=3) | 2 |
| Maddock et al. (2009) (23) | 15 | Agoraphobia (*n*=14), GAD (*n*=2), Social Phobia (*n*=2) | 2 |
| Maddock et al. (2013) (24) | 10 | Agoraphobia (*n*=9), GAD (*n*=3), Social Phobia (*n*=2) | 2 |
| Trzesniak et al. (2010) (25) | 25 | Agoraphobia (*n*=10) | 0 |
| Long et al. (2013) (26) | 11 | No information about comorbid conditions | NR |
| Pigoni et al. (2020) (11) | 11 | Confirms the absence of any comorbid conditions | 0 |
| ***SAD*** | | |  |
| Davidson et al. (1993) (30) | 20 | Avoidant Personality Disorder (*n*=8), Simple Phobia (*n*=4), Dysthymia (*n*=4), Major Depressive Disorder (*n*=1) | 1 |
| Tupler et al. (1997) (31) | 19 | Dysthymia (*n*=4), Simple Phobia (*n*=4), Dysthymia+Simple Phobia (*n*=1) | 0 |
| Phan et al. (2005) (32) | 10 | Confirms the absence of any comorbid conditions | 0 |
| Yue et al. (2012) (33) | 9 | Confirms the absence of any comorbid conditions | 0 |
| Howells et al. (2015) (34) | 18 | Confirms "no significant psychiatric comorbidity" | 0 |
| Tukel et al. (2016) (35) | 24 | Confirms the absence of any comorbid conditions | 0 |

**Table S5**. Cortical metabolite moderation by diagnostic subgroup

|  | Subgroup | K | Pts | HC | Subgroups  or Effect Size  (95% CI) | P value | Percent  Diff  Pt - HC | Heterogeneity  I^2^%, Q, P value |
| --- | --- | --- | --- | --- | --- | --- | --- | --- |
| *tCho* |  |  |  |  |  |  |  |  |
| Dx Groups | All Cortical | 16 | 234 | 244 | *SAD v GAD v PD* | .58 |  | 58.9, 31.8, .003 |
|  | excl 1° outliers* | 14 | 193 | 209 | *SAD v GAD v PD* | .57 |  | 0.0, 1.1, .44 |
|  | SAD | 5 | 80 | 72 | -.11 (+.52 to -.73) | .74 | -3.0% | 70.6, 12.1, .02 |
|  | excl 1° outlier* | 4 | 61 | 61 | -.29 (+.07 to -.65) | .11 | -5.8% | 0.0, 3.2, .36 |
|  | PD | 6 | 83 | 96 | -.30 (+.22 to -.83) | .26 | -2.2% | 65.9, 15.9, .007 |
|  | excl 1° outlier* | 5 | 61 | 71 | **-.55 (-.19 to -.90)** | **.002** | **-6.7%** | 0.0, 4.1, .39 |
|  | GAD | 5 | 73 | 79 | **-.50 (-.17 to -.83)** | **.003** | **-7.6%** | 0.0, 3.8, .44 |
| *NAA* |  |  |  |  |  |  |  |  |
| Dx Groups | All Cortical | 18 | 269 | 275 | *SAD v GAD v PD* | .56 |  | 58.4, 36.8 .001 |
|  | excl 1° outliers* | 16 | 232 | 238 | *SAD v GAD v PD* | .23 |  | 0.0, 9.1 .76 |
|  | SAD | 6 | 98 | 86 | -.17 (+.46 to -.80) | .59 | -3.5% | 76.3, 23.2 .0003 |
|  | excl 1° outlier* | 5 | 76 | 64 | **-.48 (-.14 to -.82)** | **.0055** | **-5.7%** | 0.0, 0.6, .97 |
|  | PD | 6 | 84 | 96 | -.26 (+.09 to -.61) | .14 | -2.6% | 25.9, 6.3, .28 |
|  | GAD | 6 | 87 | 93 | +.08 (-.28 to +.44) | .65 | +0.9% | 31.3, 7.3, .20 |
|  | excl 1° outlier* | 5 | 72 | 78 | -.07 (-.39 to +.25) | .67 | -2.2% | 0.0, 2.2, .69 |
| *Creatine* |  |  |  |  |  |  |  |  |
| All cortical | PD | 3 | 41 | 53 | -.02 (+.40 to +.44) | .94 |  | 4.1, 2.2, .33 |
|  | GAD | 4 | 58 | 63 | -.10 (+.33 to -.52) | .65 |  | 27.1, 4.3, .23 |
| *M-inositol* |  |  |  |  |  |  |  |  |
| All cortical | SAD | 3 | 61 | 52 | -.06 (+.37 to -.48) | .79 |  | 20.7, 2.5, .28 |
|  | GAD | 3 | 38 | 41 | -.21 (+.34 to -.77) | .45 |  | 32.8, 2.9, .23 |
| *Glx* |  |  |  |  |  |  |  |  |
| All cortical | PD | 3 | 47 | 54 | +.14 (+.61 to -.33) | .57 |  | 26.9, 2.7, .26 |

* after exclusion of datasets with an effect size that fell outside the 95% confidence limits for the primary meta-analysis of patient versus control differences, as shown in manuscript Table 1 ("primary outliers", or 1° outliers).

**Table S6**. Patient vs. control differences moderation by medication treatment status

Unmed = 100% of patients unmedicated; >50% AD = >50% of patients taking psychiatric meds, mostly antidepressants

|  | Region | K | Pts | HC | Subgroups  or Effect Size  (95% CI) | P value | Percent  Diff  Pt - HC | Heterogeneity  I^2^%, Q, P value |
| --- | --- | --- | --- | --- | --- | --- | --- | --- |
| Choline |  |  |  |  |  |  |  |  |
| Tx Groups | All Cortical | 16 | 234 | 244 | *unmed vs >50% AD* | .49 |  | 58.2, 33.4, .002 |
|  | *Excl. outliers** | 14 | 193 | 209 | *unmed vs >50% AD* | .73 |  | 6.7, 12.1, .44 |
|  | Unmed | 11 | 161 | 157 | **-.37 (-.02 to -.71)** | **.036** | **-5.8%** | 55.5, 22.0, .015 |
|  | *Excl. outlier** | 10 | 142 | 147 | **-.48 (-.23 to -.73)** | **.0002** | **-7.2%** | 10.5, 9.4, .40 |
|  | >50% AD | 5 | 73 | 87 | -.15 (+.38 to -.68) | .57 | -1.2% | 63.0, 11.4, .02 |
|  | *Excl. outlier**^a^ | 4 | 51 | 62 | **-.40 (-.02 to -.77)** | **.041** | **-5.9%** | 0.0, 2.7. .44 |
| NAA |  |  |  |  |  |  |  |  |
| Tx Groups | All Cortical | 18 | 269 | 275 | *unmed vs >50% AD* | .62 |  | 57.7, 38.3, .001 |
|  | *Excl. outliers** | 16 | 232 | 238 | *unmed vs >50% AD* | .51 |  | 22.5, 17.8, .21 |
|  | Unmed | 13 | 195 | 187 | -.07 (+.27 to -.42) | .68 | -1.5% | 63.8, 34.2 .0006 |
|  | *Excl. outliers** | 11 | 158 | 150 | **-.28 (-.05 to -.51)** | **.015** | **-4.0%** | 0.0, 8.0, .63 |
|  | >50% AD | 5 | 74 | 88 | -.23 (+.09 to -.55) | .15 | -2.3% | 1.6, 4.0, .40 |
| Creatine |  |  |  |  |  |  |  |  |
| Tx Groups | All Cortical ^b^ | 9 | 118 | 135 | **unmed** vs >50% AD | **.007** |  | 0.0, 3.5, .83 |
|  | Unmed | 5 | 58 | 62 | **-.50 (-.14 to -.87)** | **.007** | **-9.3%** | 0.0, 2.6, .63 |
|  | >50% AD | 4 | 60 | 73 | +.19 (+.53 to -.16) | .29 | +2.0% | 0.0, 1.0, .81 |
| m-Inositol |  |  |  |  |  |  |  |  |
| Tx Groups | All Cortical^b^ | 8 | 132 | 134 | unmed vs >50% AD | .79 |  | 0.0, 5.6, .47 |
|  | Unmed | 3 | 61 | 52 | -0.06 (+.37 to -.48) | .79 |  | 20.7, 2.5, .28 |
|  | >50% AD | 5 | 71 | 82 | -.14 (+.18 to -.46) | .33 |  | 0.0, 3.0, .55 |
| Glx |  |  |  |  |  |  |  |  |
| Tx Groups | All Cortical^b^ | 6 | 92 | 97 | unmed vs >50% AD | .62 |  | 32.5, 6.0, .20 |
|  | Unmed | 3 | 43 | 48 | -.21 (-.70 to +.28) | .40 |  | 24.4, 2.9, .23 |
| Lactate | All Cortical^c^ |  |  |  |  |  |  |  |
|  | Unmed | 3 | 33 | 37 | +.04 (-.44 to +.52) | .88 |  | 2.1, 2.3, .31 |
| Glutamate | All Cortical^c^ |  |  |  |  |  |  |  |
|  | Unmed | 5 | 59 | 70 | -.22 (-.69 to +.25) | .36 |  | 42.3, 6.9, .14 |
| GABA | All Cortical^c^ |  |  |  |  |  |  |  |
|  | Unmed | 3 | 42 | 39 | -.26 (+.53 to -1.04) | .52 |  | 66.3, 6.1, .048 |

* after exclusion of datasets with an effect size that fell outside the 95% confidence limits for the primary meta-analysis of patient versus control differences, as shown in manuscript Table 1 ("primary outliers", or 1° outliers).

^a^ no longer significant when excluding study only reporting lifetime diagnosis. ^b^subgroup comparison of unmedicated to >50% medicated datasets is exploratory for these metabolites, as k < 10. ^c^all lactate, glutamate and GABA datasets were unmedicated.

**Table S7**. Other moderators: Normalization (water vs Cr), field strength (< 3 Tesla vs ≥ 3 Tesla, % male,

age, log echo time, and study quality rating

|  | Region | K | Pts | HC | Subgroups  or Effect Size  (95% CI) | P value | Percent  Diff ^a^  Pt - HC | Heterogeneity  I^2^%, Q, P value |
| --- | --- | --- | --- | --- | --- | --- | --- | --- |
| Choline |  |  |  |  |  |  |  |  |
| W vs Cr | All Cortical | 14 | 203 | 213 | *Water vs. creatine* | .51 |  | 59.8, 29.6, .003 |
|  | *Excl. outliers** | 12 | 162 | 178 | *Water vs. creatine* | .61 |  | 8.8, 10.5, .39 |
|  | Water | 5 | 67 | 81 | -.08 (+.41 to -.57) | .74 | -1.2% | 53.0, 8.7, .07 |
|  | *Excl. outlier** | 4 | 45 | 56 | -.31 (-.71 to +.09) | .12 | -3.1% | 0.0, 1.6, .67 |
|  | Creatine | 9 | 136 | 132 | -.32 (+.10 to -.73) | .13 | -4.0% | 63.3, 20.9, .007 |
|  | *Excl. outlier** | 8 | 117 | 122 | **-.45 (-.15 to -.75)** | **.003** | **-5.6%** | 22.9, 9.0, .25 |
| NAA |  |  |  |  |  |  |  |  |
| W vs Cr | All Cortical | 16 | 238 | 244 | *Water vs. creatine* | .26 |  | 58.7, 34.3, .002 |
|  | *Excl. outliers** | 14 | 201 | 207 | *Water vs. creatine* | .54 |  | 0.0, 10.8, .55 |
|  | Water | 5 | 68 | 82 | **-.36 (-.03 to -.68)** | **.033** | **-3.5%** | 0.0, 1.6, .81 |
|  | Creatine | 11 | 170 | 162 | +.00 (+.40 to -.39) | .98 | -0.5% | 68.5, 32.7 .0003 |
|  | *Excl. outliers** | 9 | 133 | 125 | -.23 (-.51 to +.04) | .10 | -2.9% | 17.4, 9.2, .33 |
| Choline |  |  |  |  |  |  |  |  |
| Field Strength | All Cortical | 16 | 234 | 244 | *<3T vs. ≥3T* | .66 |  | 59.7, 35.0, .001 |
|  | *Excl. outliers** | 14 | 193 | 209 | *<3T vs. ≥3T* | .52 |  | 2.9, 11.8, .46 |
| % Male | All Cortical | 16 | 234 | 244 | *% male* | .87 |  | 60.0, 35.1 .001 |
|  | *Excl. outliers** | 14 | 193 | 209 | *% male* | .56 |  | 6.6, 11.9, .46 |
| Patient Age | All Cortical | 14 | 213 | 212 | *Mean Pt. age* | .66 |  | 64.5, 34.0 .0007 |
|  | *Excl. outliers** | 12 | 172 | 177 | *Mean Pt. age* | .61 |  | 5.9, 10.3, .41 |
| log TE | All Cortical | 16 | 234 | 244 | *log TE* | .069^1^ |  | 49.0, 26.7, .016 |
|  | *Excl. outliers** | 14 | 193 | 209 | *log TE* | .16 |  | 0.0, 10.2, .60 |
| *Low COV group* | All Cortical | 8 | 100 | 116 | *log TE* | .93 |  | 0.0, 9.6, .47 |
| Study Quality | All Cortical | 16 | 234 | 244 | ***Quality Rating*** | **.037** |  | 47.1, 26.5, .02 |
|  | *Excl. outliers** | 14 | 193 | 209 | *Quality Rating* | .27 |  | 2.1, 11.0, .53 |
| NAA |  |  |  |  |  |  |  |  |
| Field Strength | All Cortical | 18 | 269 | 275 | *<3T vs. ≥3T* | .49 |  | 57.3, 37.9, .002 |
|  | *Excl. outliers** | 16 | 232 | 238 | *<3T vs. ≥3T* | .51 |  | 0.0, 11.6, .63 |
| % Male | All Cortical | 18 | 269 | 275 | *% male* | .43 |  | 57.0, 37.9, .002 |
|  | *Excl. outliers** | 16 | 232 | 238 | *% male* | .97 |  | 0.0, 12.5, .56 |
| Patient Age | All Cortical | 16 | 248 | 243 | *Mean Pt. age* | .66 |  | 60.8, 36.2, .001 |
|  | *Excl. outliers** | 14 | 211 | 206 | *Mean Pt. age* | .94 |  | 0.0, 11.2, .51 |
| log TE | All Cortical | 18 | 269 | 275 | *log TE* | .93 |  | 58.4, 39.2, .001 |
|  | *Excl. outliers** | 16 | 232 | 238 | *log TE* | .97 |  | 0.0, 12.5, .56 |
| Study Quality | All Cortical | 18 | 269 | 275 | *Quality Rating* | .28 |  | 54.4, 35.1, .004 |
|  | *Excl. outliers** | 16 | 232 | 238 | *Quality Rating* | .32 |  | 0.0, 11.1, .68 |

* after exclusion of datasets with an effect size that fell outside the 95% confidence limits for the primary meta-analysis of patient versus control differences, as shown in manuscript Table 1 (("primary outliers", or 1° outliers).

^1^ Non-significant trend for larger reductions in cortical tCho in datasets using longer TEs.

**Figure S2 - Forest plots for tCho from additional brain regions**

SAD = Social Anxiety Disorder; GAD = Generalized Anxiety Disorder; PD = Panic Disorder; ACC = Anterior Cingulate Cortex; Put = Putamen; L = left; BG = Basal Ganglia; HC = Hippocampus; R = right

**Anterior Cingulate Cortex tCho, K = 7 studies**


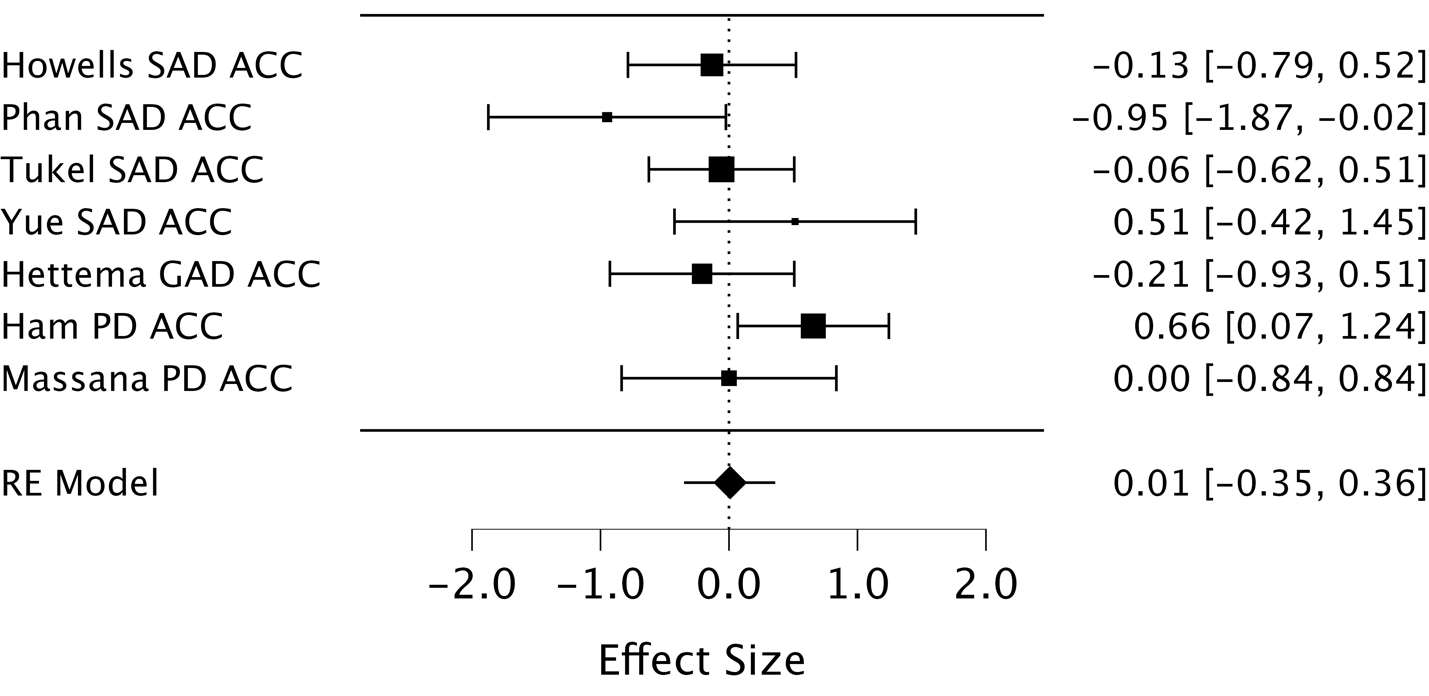


**Basal Ganglia tCho, K = 4 studies**


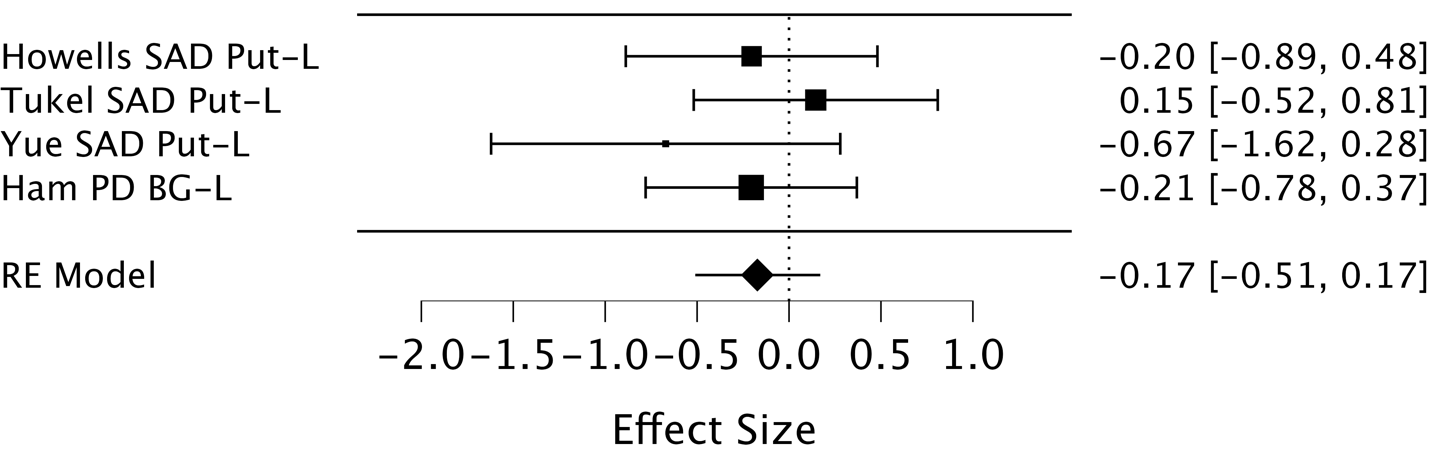


**Hippocampus tCho, K = 3 studies**


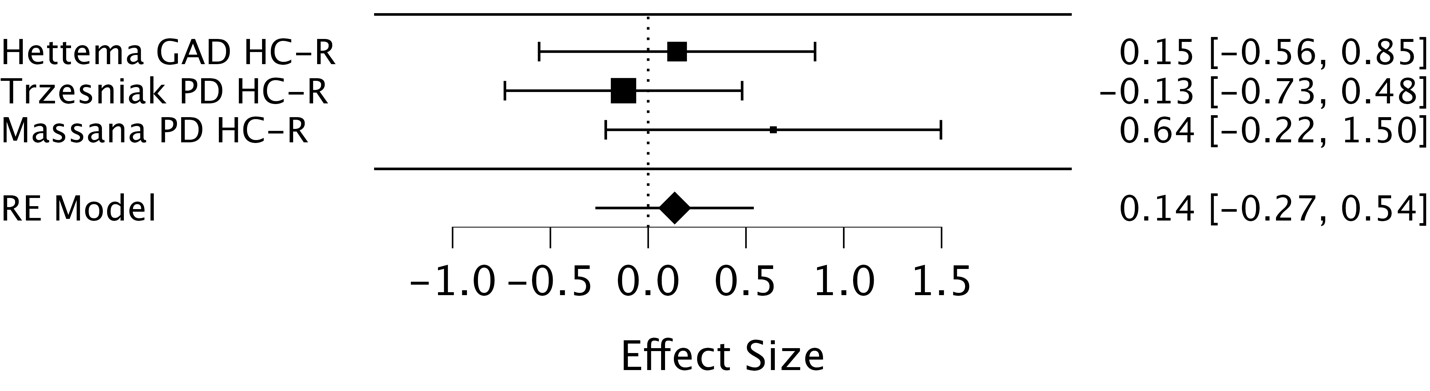


**Figure S3 - Forest plots for NAA from additional brain regions**

SAD = Social Anxiety Disorder; GAD = Generalized Anxiety Disorder; PD = Panic Disorder; ACC = Anterior Cingulate Cortex; PFC = Prefrontal Cortex; OC = Occipital Cortex; WB-W = whole brain white matter; CS = Centrum Semiovale; B = bilateral; L = left; R = right; Put = Putamen; Caud = Caudate; BG = Basal Ganglia; HC = Hippocampus

**Anterior Cingulate Cortex NAA, K = 7 studies**


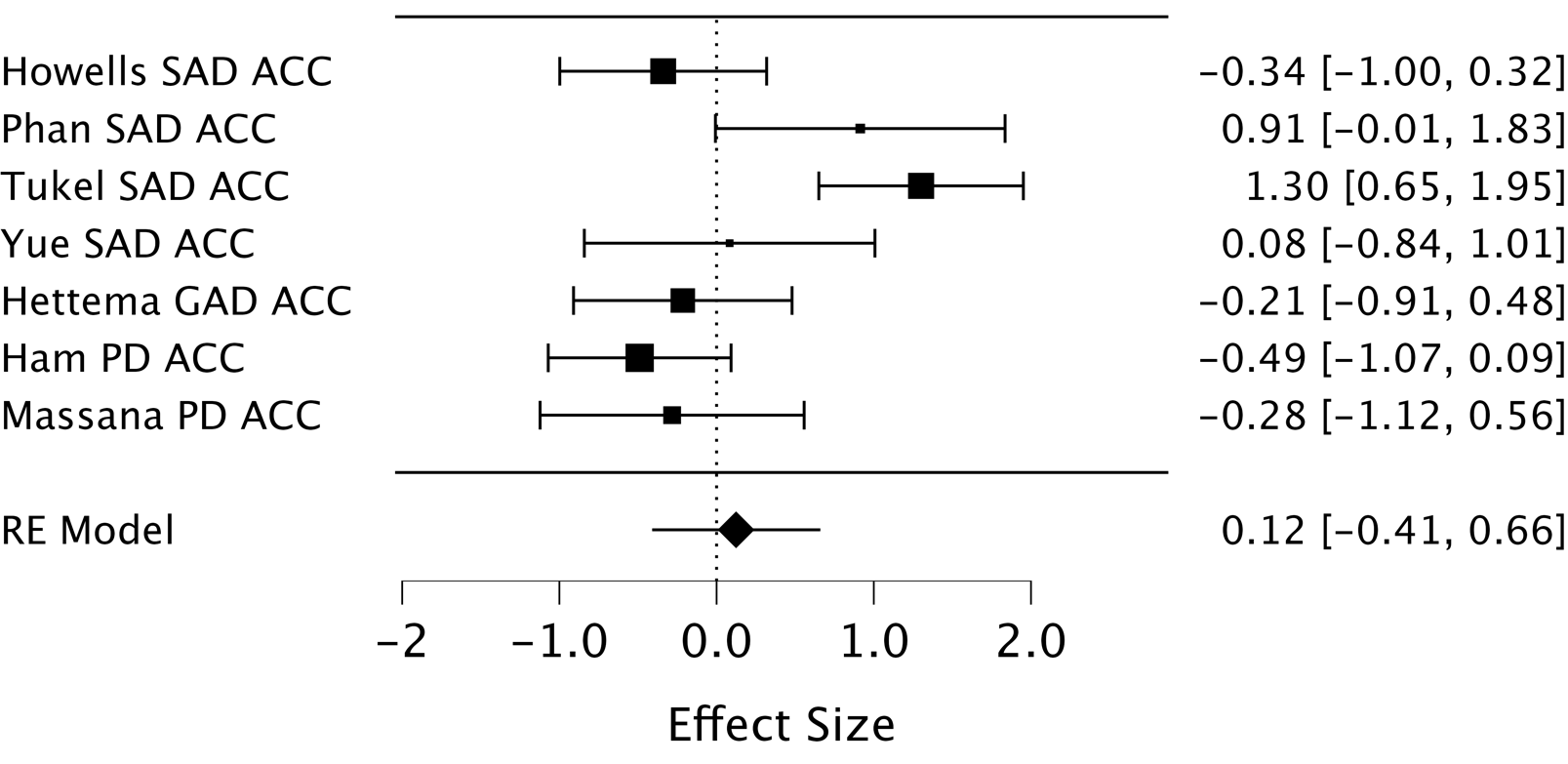


#### Prefrontal Cortex NAA, K = 7 studies


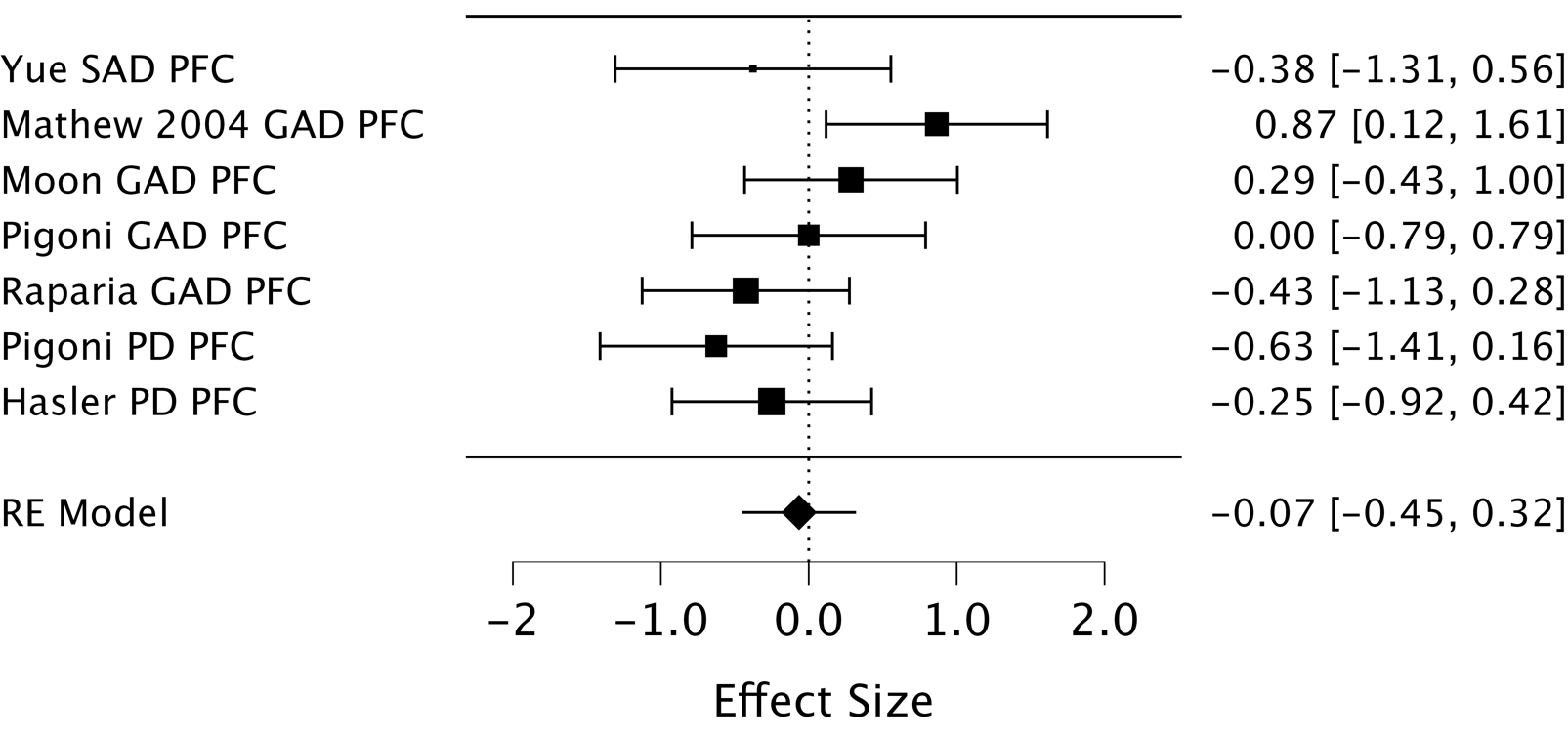


#### Occipital Cortex NAA, K = 3 studies


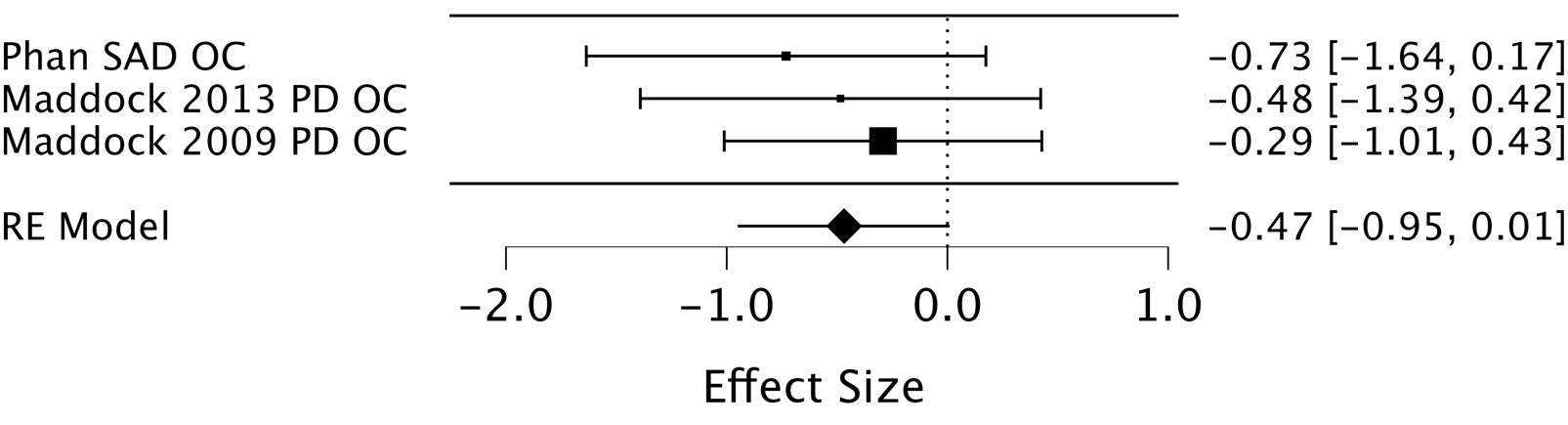


#### White Matter NAA, K = 3 studies


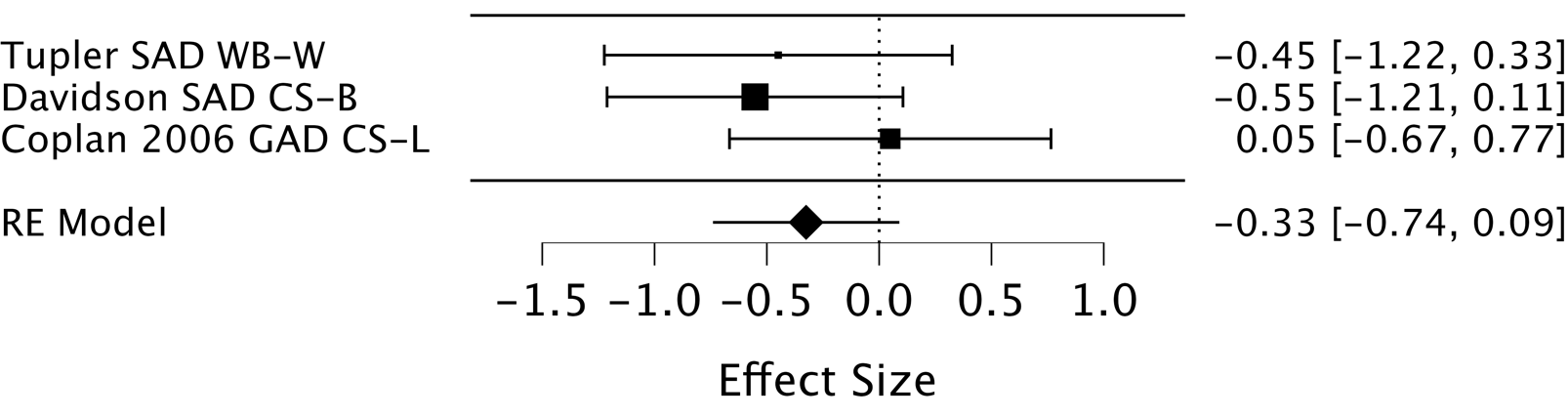


**Basal Ganglia NAA, K = 5 studies**


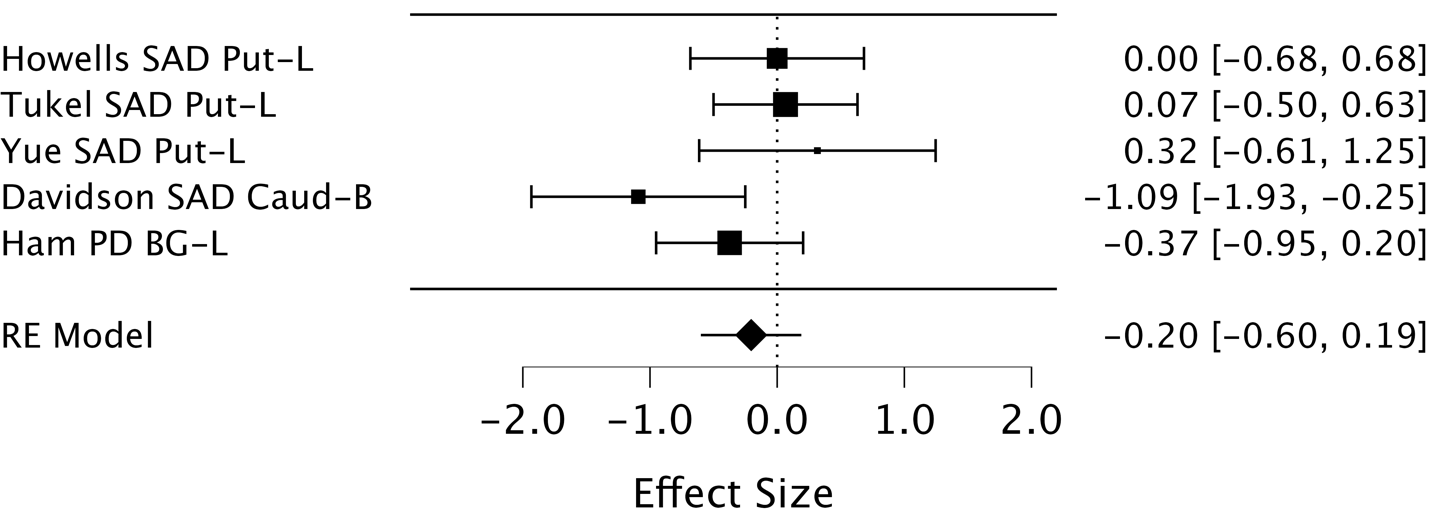


**Hippocampus NAA, K = 4 studies**


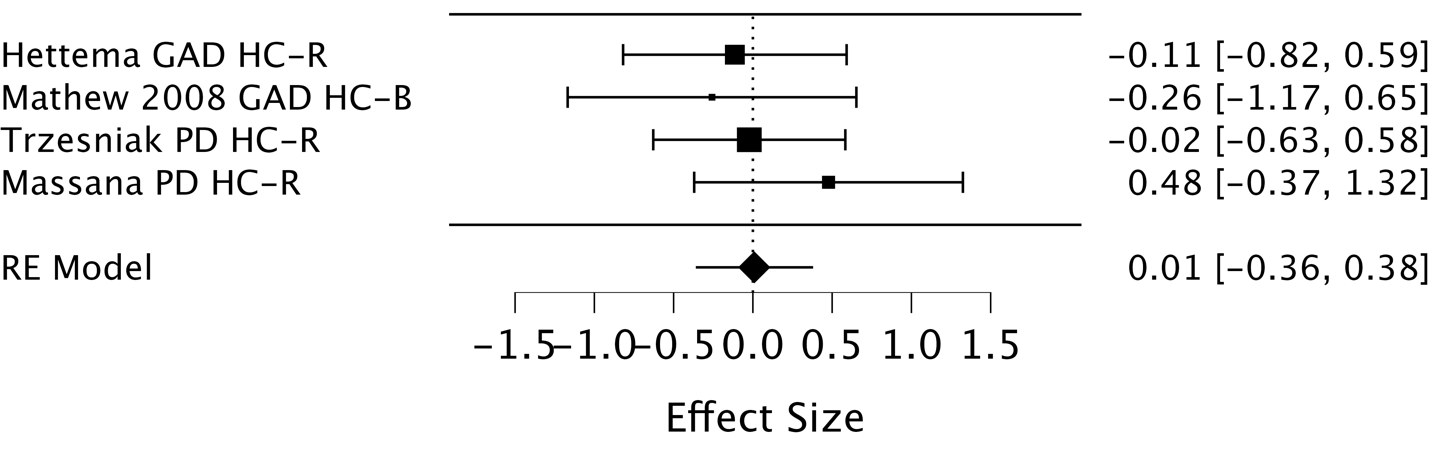


**Figure S4 - Forest plots for total Creatine**

SAD = Social Anxiety Disorder; GAD = Generalized Anxiety Disorder; PD = Panic Disorder; ACC = Anterior Cingulate Cortex; PFC = Prefrontal Cortex; OC = Occipital Cortex; CS = Centrum Semiovale; PMC = Premotor Cortex; L = left; R = right

**Cortical Creatine, k = 9 studies**


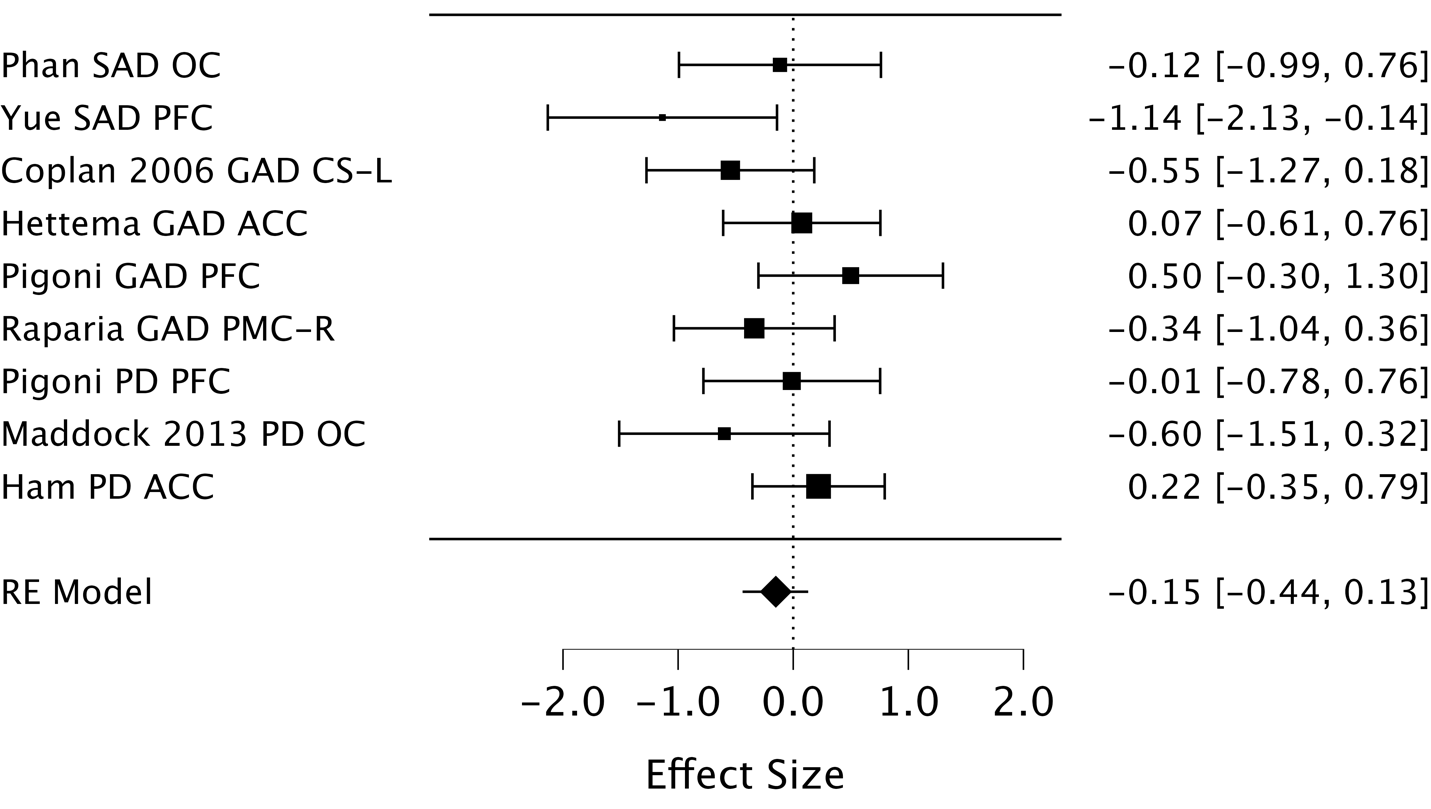


#### ACC Creatine, k = 4 studies


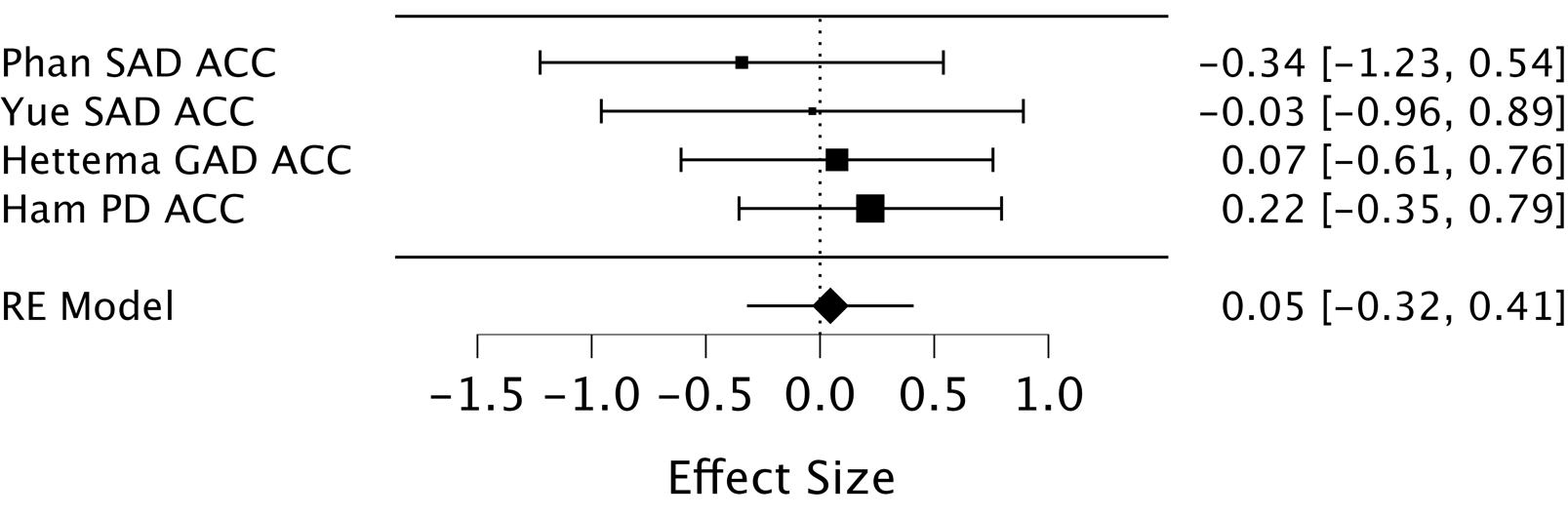


#### PFC Creatine, k = 4 studies


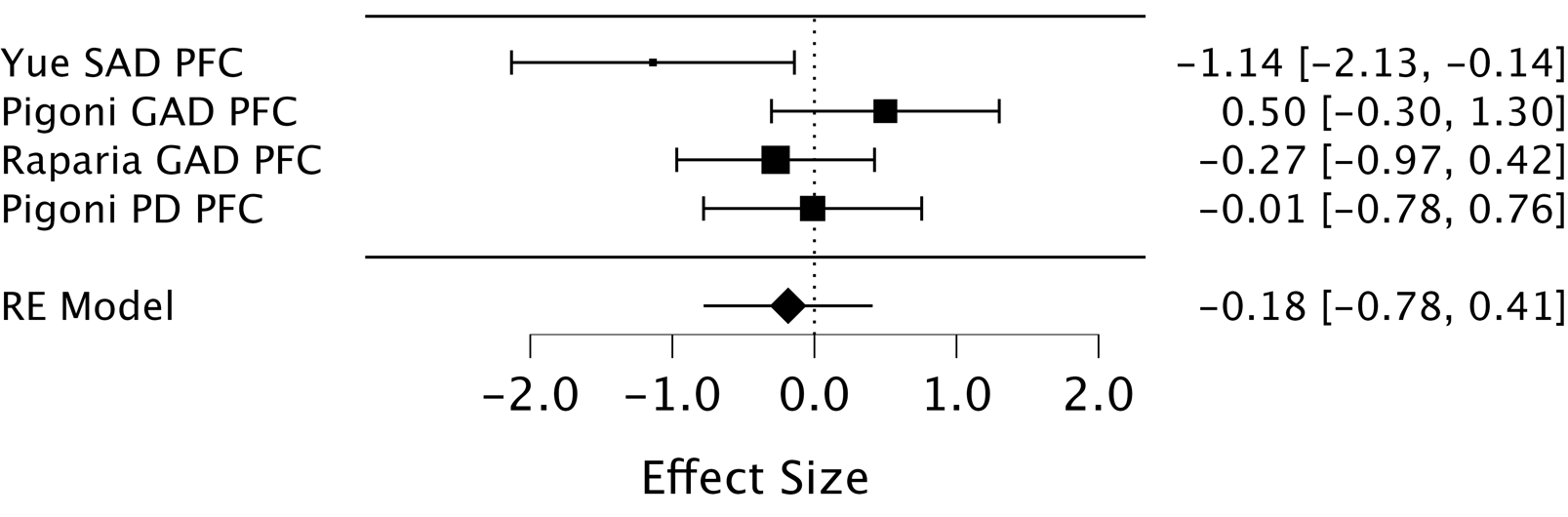


**Figure S5 - Forest plots for Myo-Inositol**

SAD = Social Anxiety Disorder; GAD = Generalized Anxiety Disorder; PD = Panic Disorder; ACC = Anterior Cingulate Cortex; PFC = Prefrontal Cortex; WB-G = whole brain gray matter

**Cortical Myo-Inositol, k = 8 studies**


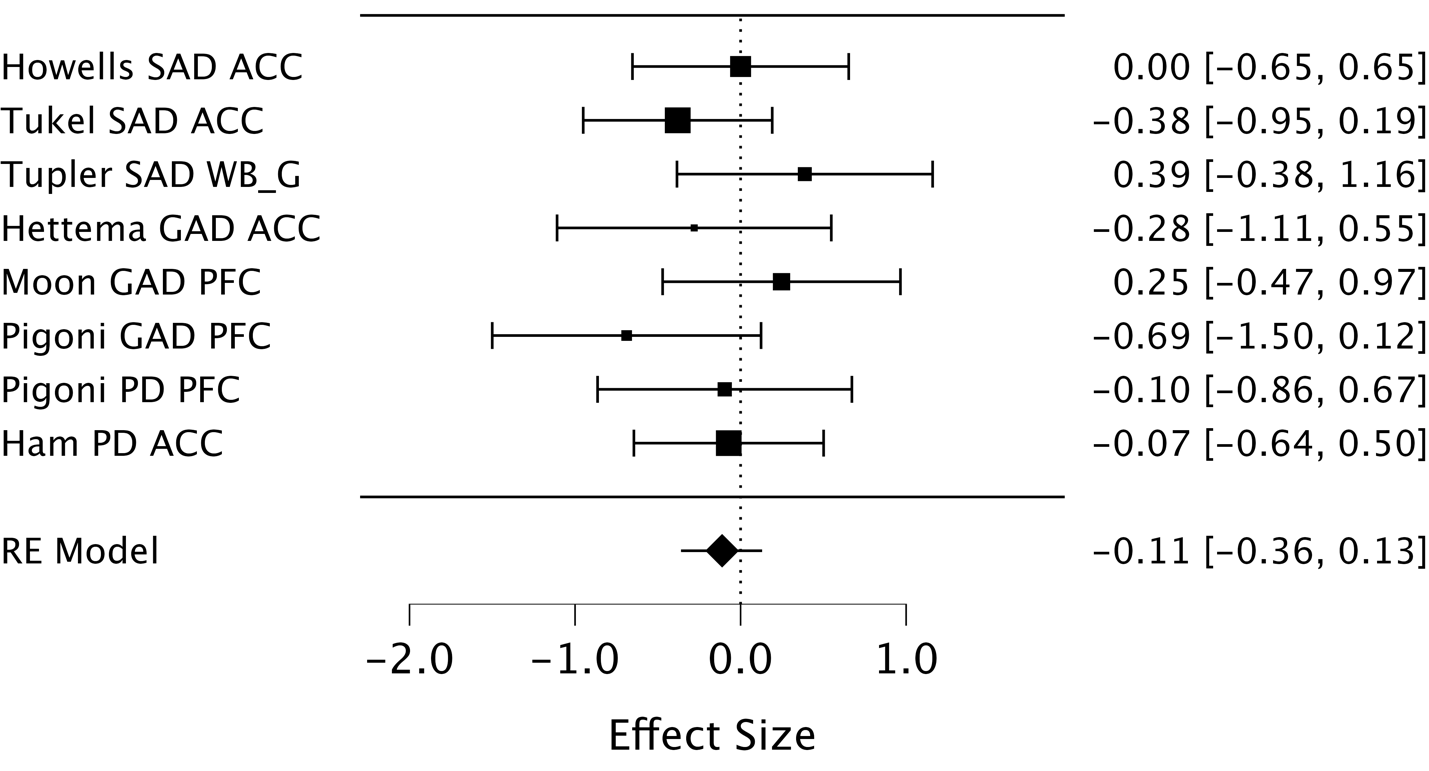


**ACC Myo-Inositol, k = 4 studies**
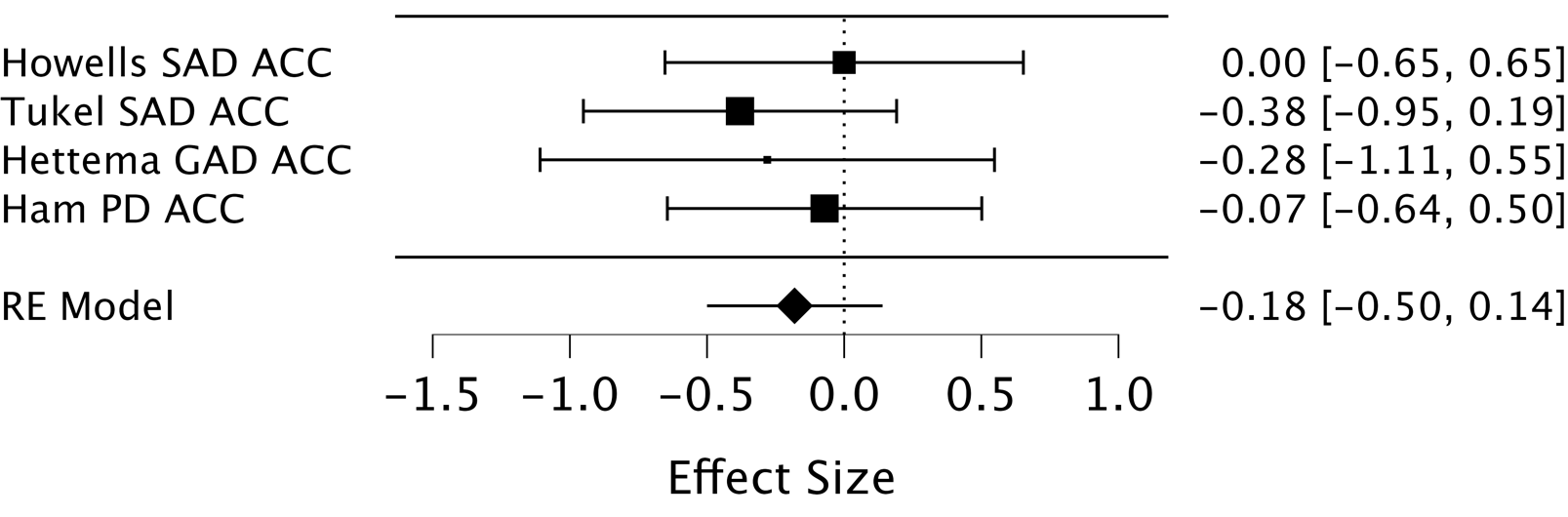


**Prefrontal Cortex Myo-Inositol, k = 3 studies**


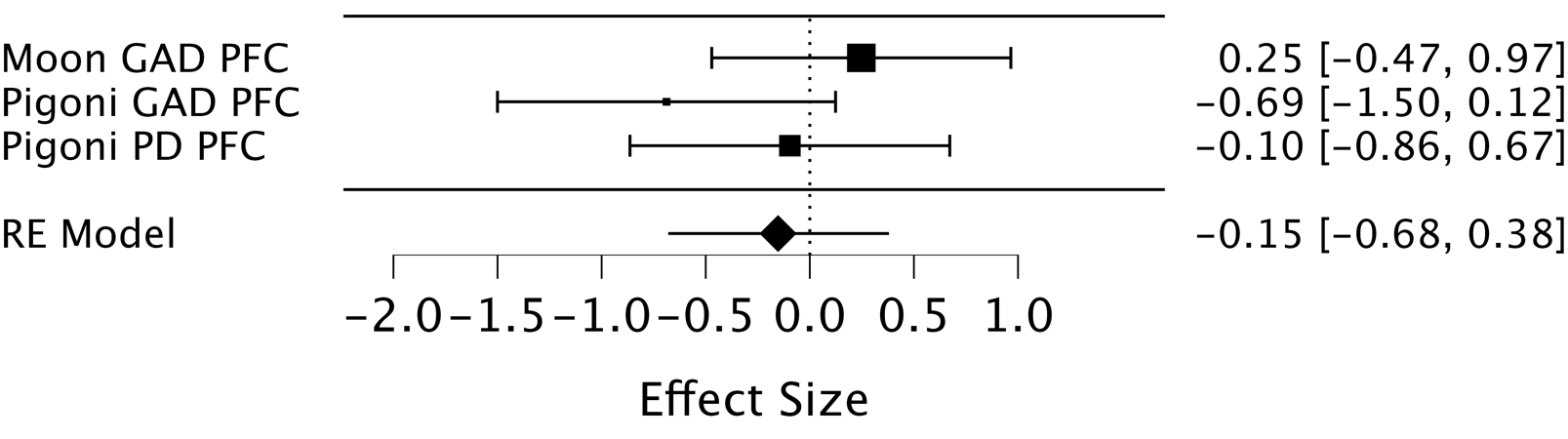


**Figure S6 - Forest plots for Glutamate**

SAD = Social Anxiety Disorder; GAD = Generalized Anxiety Disorder; PD = Panic Disorder; ACC = Anterior Cingulate Cortex; PFC = Prefrontal Cortex; OC = Occipital Cortex

**Cortical Glutamate, k = 5 studies**


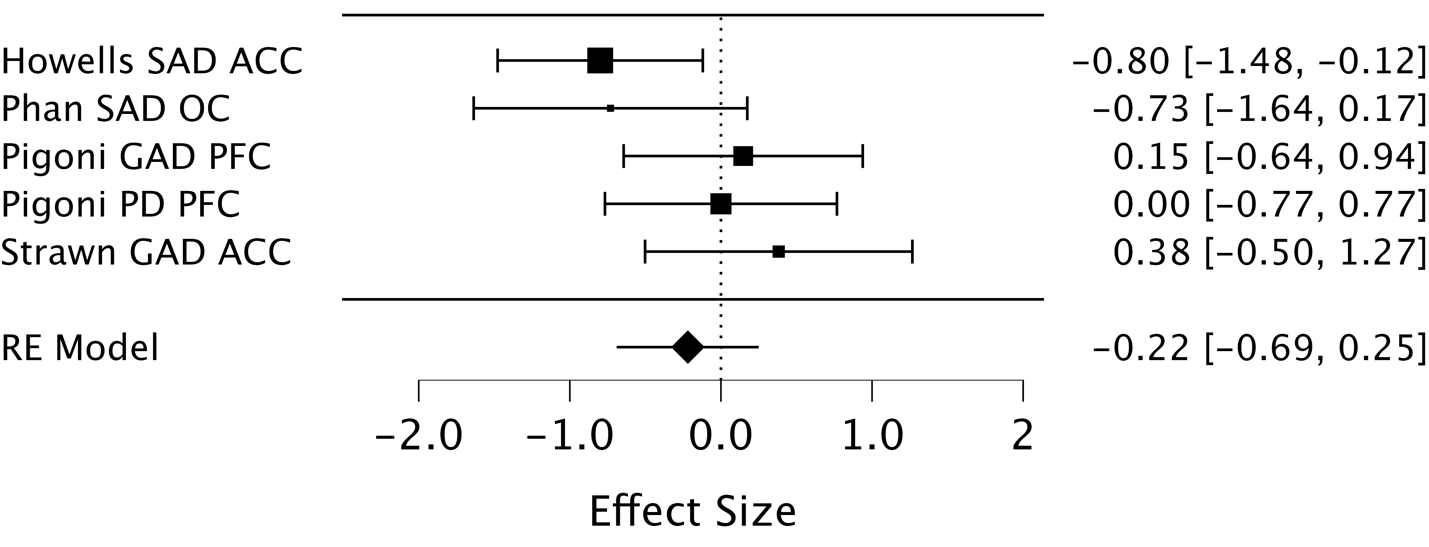


**ACC Glutamate, k = 5 studies**


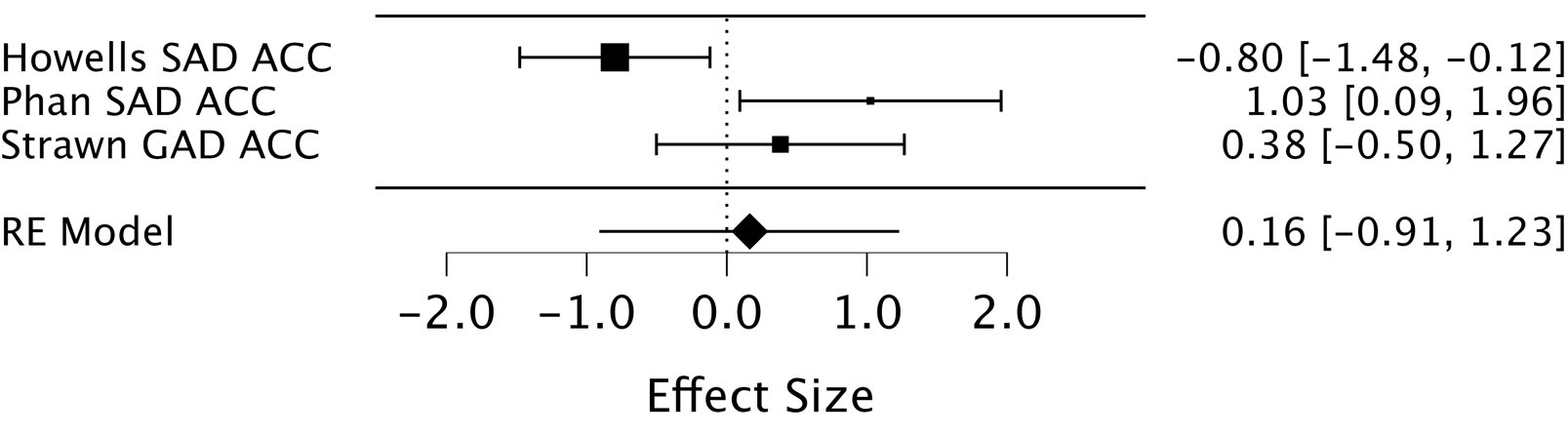


**Figure S7 - Forest plots for Glx**

SAD = Social Anxiety Disorder; GAD = Generalized Anxiety Disorder; PD = Panic Disorder; ACC = Anterior Cingulate Cortex; PFC = Prefrontal Cortex; OC = Occipital Cortex

**Cortical Glx, k = 6 studies**


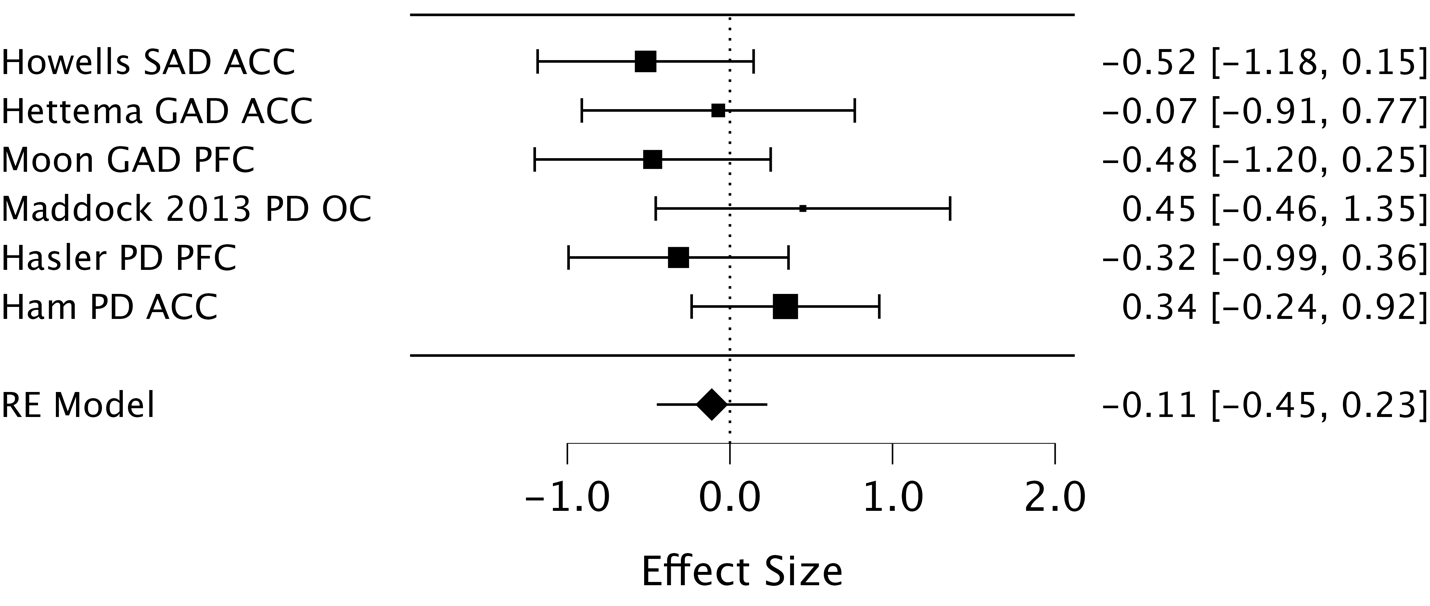


**ACC Glx, k = 3 studies**


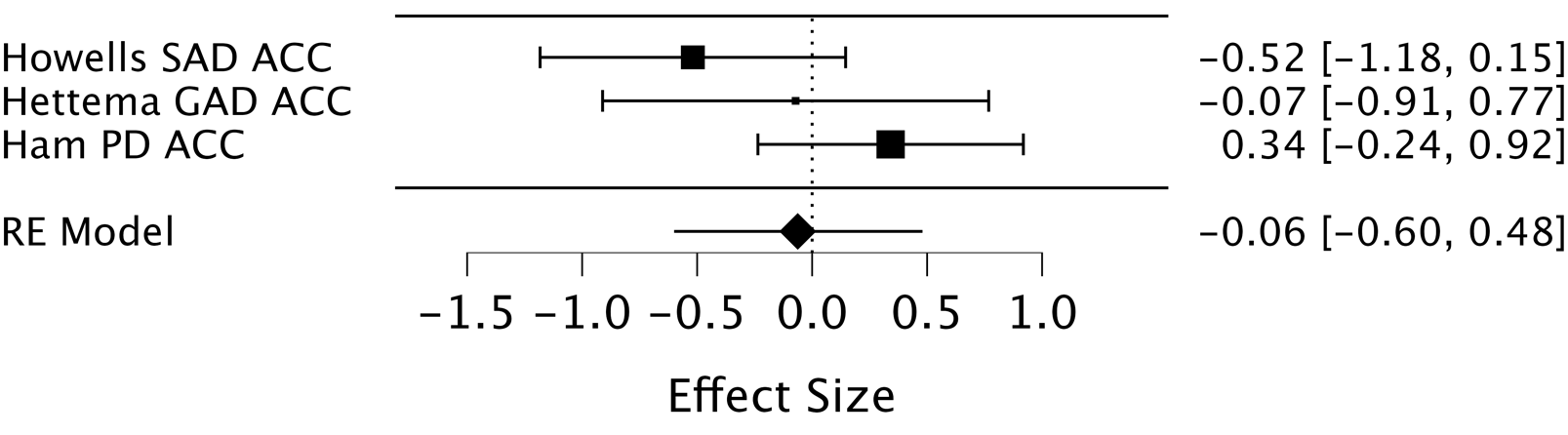


**Figure S8 - Forest plots for GABA and Lactate**

PD = Panic Disorder; PFC = Prefrontal Cortex; OC = Occipital Cortex

**Cortical GABA, k = 3, all in PD patients**


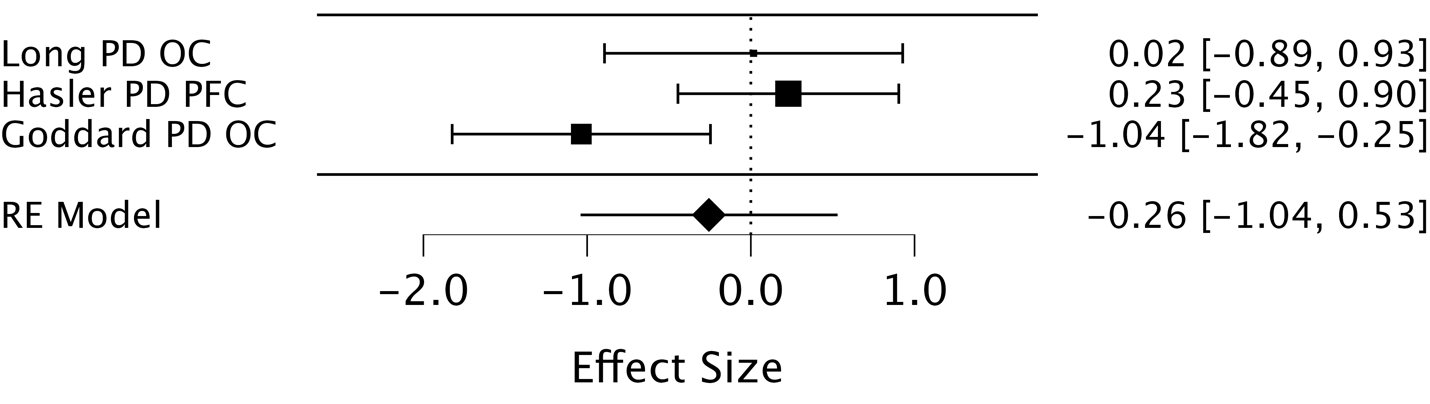


**Cortical Lactate, k = 3 studies, all in PD patients**


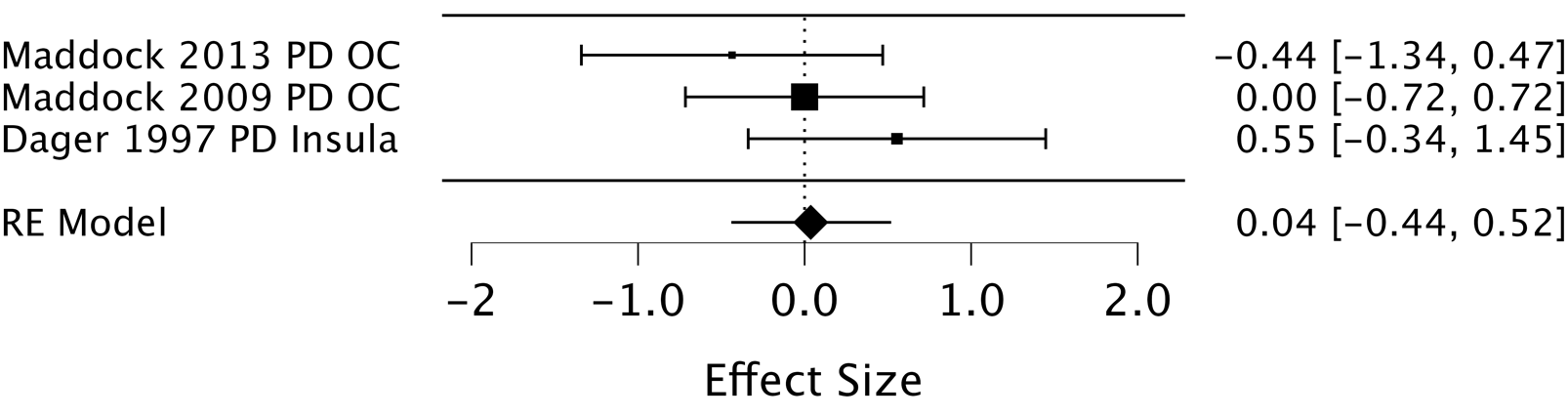


**Supplemental Results for primary meta-analyses of other metabolites:**

***Creatine, myo-inositol, glutamate, glx, GABA, and lactate***

*Creatine and phosphocreatine (tCr)*

All regional results for tCr are summarized in Table 1. Four datasets reporting tCr were available for both the ACC and the PFC. No significant or trend-level changes were observed in either region. Nine datasets reported on tCr measured in at least one cortical region. Four datasets reported on more than one cortical region. For these, only the cortical region with the lowest COV for creatine was included. The 9 analyzed regions included 2 ACC, 3 PFC, 2 occipital, 1 premotor, and 1 centrum semiovale. No significant difference in tCr levels was seen across these cortical measurements. Importantly, only one of the studies reporting tCr levels normalized tCr to a CSF-corrected water signal (2 used phantom replacement and 6 used uncorrected water).

*Myo-Inositol*

All regional results for myo-inositol are summarized in Table 1. Myo-inositol levels were available from four datasets in the ACC and three datasets in the PFC. No significant or trend-level changes were observed in either region. Eight datasets reported on myo-inositol measured in at least one cortical region. Two datasets reported on more than one cortical region. For these, only the cortical region with the lowest COV for myo-inositol was included. The eight analyzed regions included 4 ACC, 3 PFC, and 1 overall cortical gray matter. No significant difference in myo-inositol levels was seen across these cortical measurements.

*Glutamate*

Five studies reported on glutamate levels. Of these, three reported values for the ACC, two for the PFC, and one for the occipital cortex. One study reported values for two regions (ACC and occipital). In this case, the occipital region was included in the all-cortical analysis based on a lower mean COV. No significant difference in glutamate levels was seen across the three ACC datasets or across the five cortical datasets (Table 1).

*Glutamate plus Glutamine (Glx)*

Six studies reported on glx levels. Of these, four reported values for the ACC, one reported values for the PFC, and one reported values for the occipital cortex. No significant difference in glx levels was seen across the four ACC datasets or across the six cortical datasets (Table 1).

*GABA*

Three datasets reported GABA levels acquired with validated spectral editing methods and were included. All three datasets were from studies of patients with panic disorder. No brain region was represented by more than 2 datasets. Two datasets reported GABA levels in more than one cortical region. Of these, only the cortical region with the lowest COV for GABA was included in the analysis, which included two datasets from the occipital cortex and one dataset from the PFC. No significant difference in GABA levels was seen across these three cortical datasets (Table 1).

*Lactate*

Three datasets reporting on resting lactate levels using spectral editing sequences or echo times approximating integral multiples of the j-coupling frequency of lactate were included. All were from studies of panic disorder patients. Two reported lactate levels in the occipital cortex and one reported lactate levels in the insula. No significant difference in resting lactate levels was seen across these three cortical datasets (Table 1).

**Supplemental Results for moderating effects of diagnosis**

***Cortical Creatine, Myo-inositol, Glutamate, Glx, GABA, and Lactate***

Of the 9 datasets reporting cortical creatine levels, two studied SAD, three studied PD and four studied GAD. Of the 8 datasets reporting on cortical myo-inositol, 3 studied SAD, 2 studied GAD, and 3 studied PD. Of the 5 datasets reporting on cortical glutamate, 2 studied SAD, 2 studied GAD, and 1 studied PD. Of the 6 datasets reporting on cortical glx, 1 studied SAD, 2 studied GAD, and 3 studied PD. All 3 cortical GABA datasets and all 3 cortical lactate datasets studied people with PD. As none of these cortical metabolites were reported on by at least ten datasets, meta-analyses comparing the effects of specific diagnosis were not performed. All meta-analyses of diagnostic subgroups with k ≥ 3 were non-significant for all these metabolites, and the results are shown in Supplemental Table S3.

**Supplemental Results regarding extreme outlier cortical NAA value:**

When including all 6 studies reporting cortical NAA in SAD, no significant effect was observed (Table S3). However, one of the studies was an extreme statistical outlier in the overall analysis of cortical NAA across all three disorders (35). The effect size for this study was g = +1.30 (+.65 to + 1.95) compared to the pooled effect size of the other 17 datasets in this overall analysis of g = -0.20 (-0.40 to 0.00). When this study was excluded from the analysis of cortical NAA in SAD, the results became highly significant (k = 5, 76 Pts, 64 HCs, *g* = -0.48, 95% CI = -0.14 to -.82, p = .006) (Table S3). Since in this case, removing an extreme statistical outlier had a substantial impact on the meta-analytic results, possible reasons for the outlier value were further examined. We noted that the coefficient of variation (COV = SD/mean) for the outlier NAA value was 2.6 times lower than the next highest NAA COV value from any of the 47 other regional NAA measurements reported by the other 25 included datasets. Indeed, it was also 2.2 times lower than the lowest COV value from 154 regional NAA measurements reported in our earlier meta-analysis of schizophrenia studies (2). The low degree of variation associated with the NAA values from this study is implausible. Another anomalous feature of these data is that the authors reported using a t-test to compare 24 patient NAA values to 24 control NAA values. They reported, however, "42.32" degrees of freedom associated with the t value for this analysis, and fractional df values are not a feature of t-tests. The corresponding author of the outlier study was contacted with a request for clarification of these anomalous data, but did not respond.

References for Supplemental Materials

1. Smucny J, Carter CS, Maddock RJ. Medial Prefrontal Cortex Glutamate Is Reduced in Schizophrenia and Moderated by Measurement Quality: A Meta-analysis of Proton Magnetic Resonance Spectroscopy Studies. Biol Psychiatry. 2021 Nov 1;90(9):643–51.

2. Yang YS, Smucny J, Zhang H, Maddock RJ. Meta-analytic evidence of elevated choline, reduced N-acetylaspartate, and normal creatine in schizophrenia and their moderation by measurement quality, echo time, and medication status. NeuroImage Clin. 2023;39:103461.

3. Mathew SJ, Mao X, Coplan JD, Smith ELP, Sackeim HA, Gorman JM, et al. Dorsolateral Prefrontal Cortical Pathology in Generalized Anxiety Disorder: A Proton Magnetic Resonance Spectroscopic Imaging Study. Am J Psychiatry. 2004 Jun 1;161(6):1119–21.

4. Mathew SJ, Price RB, Mao X, Smith ELP, Coplan JD, Charney DS, et al. Hippocampal N-Acetylaspartate Concentration and Response to Riluzole in Generalized Anxiety Disorder. Biol Psychiatry. 2008 May;63(9):891–8.

5. Coplan JD, Mathew SJ, Mao X, Smith ELP, Hof PR, Coplan PM, et al. Decreased choline and creatine concentrations in centrum semiovale in patients with generalized anxiety disorder: Relationship to IQ and early trauma. Psychiatry Res Neuroimaging. 2006 Jun;147(1):27–39.

6. Coplan JD, Webler R, Gopinath S, Abdallah CG, Mathew SJ. Neurobiology of the dorsolateral prefrontal cortex in GAD: Aberrant neurometabolic correlation to hippocampus and relationship to anxiety sensitivity and IQ. J Affect Disord. 2018 Mar;229:1–13.

7. Hettema JM, Kettenmann B, Ahluwalia V, McCarthy C, Kates WR, Schmitt JE, et al. Pilot multimodal twin imaging study of generalized anxiety disorder: Twin GAD MRI. Depress Anxiety. 2012 Mar;29(3):202–9.

8. Strawn JR, Chu WJ, Whitsel RM, Weber WA, Norris MM, Adler CM, et al. A Pilot Study of Anterior Cingulate Cortex Neurochemistry in Adolescents with Generalized Anxiety Disorder. Neuropsychobiology. 2013;67(4):224–9.

9. Moon C, Kang H, Jeong G. Metabolic change in the right dorsolateral prefrontal cortex and its correlation with symptom severity in patients with generalized anxiety disorder: Proton magnetic resonance spectroscopy at 3 T esla. Psychiatry Clin Neurosci. 2015 Jul;69(7):422–30.

10. Raparia E, Coplan JD, Abdallah CG, Hof PR, Mao X, Mathew SJ, et al. Impact of childhood emotional abuse on neocortical neurometabolites and complex emotional processing in patients with generalized anxiety disorder. J Affect Disord. 2016 Jan;190:414–23.

11. Pigoni A, Delvecchio G, Squarcina L, Bonivento C, Girardi P, Finos L, et al. Sex differences in brain metabolites in anxiety and mood disorders. Psychiatry Res Neuroimaging. 2020 Nov;305:111196.

12. Mathew SJ, Mao X, Keegan KA, Levine SM, Smith ELP, Heier LA, et al. Ventricular cerebrospinal fluid lactate is increased in chronic fatigue syndrome compared with generalized anxiety disorder: an *in vivo* 3.0 T^1^ H MRS imaging study. NMR Biomed. 2009 Apr;22(3):251–8.

13. Abdallah CG, Coplan JD, Jackowski A, Sato JR, Mao X, Shungu DC, et al. Riluzole effect on occipital cortex: A structural and spectroscopy pilot study. Neurosci Lett. 2012 Nov;530(1):103–7.

14. Abdallah CG, Coplan JD, Jackowski A, Sato JR, Mao X, Shungu DC, et al. A pilot study of hippocampal volume and N-acetylaspartate (NAA) as response biomarkers in riluzole-treated patients with GAD. Eur Neuropsychopharmacol. 2013 Apr;23(4):276–84.

15. Coplan JD, Fathy HM, Abdallah CG, Ragab SA, Kral JG, Mao X, et al. Reduced hippocampal N-acetyl-aspartate (NAA) as a biomarker for overweight. NeuroImage Clin. 2014;4:326–35.

16. Moon CM, Jeong GW. Brain morphological alterations and cellular metabolic changes in patients with generalized anxiety disorder: A combined DARTEL-based VBM and 1H-MRS study. Magn Reson Imaging. 2016 May;34(4):429–36.

17. Moon CM, Sundaram T, Choi NG, Jeong GW. Working memory dysfunction associated with brain functional deficits and cellular metabolic changes in patients with generalized anxiety disorder. Psychiatry Res Neuroimaging. 2016 Aug;254:137–44.

18. Dager SR, Richards T, Strauss W, Artru A. Single-voxel 1H-MRS investigation of brain metabolic changes during lactate-induced panic. Psychiatry Res Neuroimaging. 1997 Dec;76(2–3):89–99.

19. Goddard AW, Mason GF, Almai A, Rothman DL, Behar KL, Petroff OAC, et al. Reductions in Occipital Cortex GABA Levels in Panic Disorder Detected With 1H-Magnetic Resonance Spectroscopy. Arch Gen Psychiatry. 2001 Jun 1;58(6):556–61.

20. Massana G, Gastó C, Junqué C, Mercader JM, Gómez B, Massana J, et al. Reduced Levels of Creatine in the Right Medial Temporal Lobe Region of Panic Disorder Patients Detected with 1H Magnetic Resonance Spectroscopy. NeuroImage. 2002 Jul;16(3):836–42.

21. Ham BJ, Sung Y, Kim N, Kim SJ, Kim JE, Kim DJ, et al. Decreased GABA levels in anterior cingulate and basal ganglia in medicated subjects with panic disorder: A proton magnetic resonance spectroscopy (1H-MRS) study. Prog Neuropsychopharmacol Biol Psychiatry. 2007 Mar;31(2):403–11.

22. Hasler G, Van Der Veen JW, Geraci M, Shen J, Pine D, Drevets WC. Prefrontal Cortical Gamma-Aminobutyric Acid Levels in Panic Disorder Determined by Proton Magnetic Resonance Spectroscopy. Biol Psychiatry. 2009 Feb;65(3):273–5.

23. Maddock RJ, Buonocore MH, Copeland LE, Richards AL. Elevated brain lactate responses to neural activation in panic disorder: a dynamic 1H-MRS study. Mol Psychiatry. 2009 May;14(5):537–45.

24. Maddock RJ, Buonocore MH, Miller AR, Yoon JH, Soosman SK, Unruh AM. Abnormal Activity-Dependent Brain Lactate and Glutamate+Glutamine Responses in Panic Disorder. Biol Psychiatry. 2013 Jun;73(11):1111–9.

25. Trzesniak C, Uchida RR, Araújo D, Guimarães FS, Freitas-Ferrari MC, Filho AS, et al. 1H magnetic resonance spectroscopy imaging of the hippocampus in patients with panic disorder. Psychiatry Res Neuroimaging. 2010 Jun;182(3):261–5.

26. Long Z, Medlock C, Dzemidzic M, Shin YW, Goddard AW, Dydak U. Decreased GABA levels in anterior cingulate cortex/medial prefrontal cortex in panic disorder. Prog Neuropsychopharmacol Biol Psychiatry. 2013 Jul;44:131–5.

27. Dager D Stephen R, Marro KI, Richards TL, Metzger GD. Preliminary application of magnetic resonance spectroscopy to investigate lactate-induced panic. Am J Psychiatry. 1994 Jan 1;151(1):57–63.

28. Dager S, Strauss W, Marro K, Richards T, Metzger G, Artru A. Proton magnetic resonance spectroscopy investigation of hyperventilation in subjects with panic disorder and comparison subjects. Am J Psychiatry. 1995 May 1;152(5):666–72.

29. Dager SR, Friedman SD, Heide A, Layton ME, Richards T, Artru A, et al. Two-dimensional Proton Echo-Planar Spectroscopic Imaging of Brain Metabolic Changes During Lactate-Induced Panic. Arch Gen Psychiatry. 1999 Jan 1;56(1):70.

30. Davidson JR, Krishnan KR, Charles HC, Boyko O, Potts NL, Ford SM, et al. Magnetic resonance spectroscopy in social phobia: preliminary findings. J Clin Psychiatry. 1993 Dec;54 Suppl:19–25.

31. Tupler LA, Davidson JRT, Smith RD, Lazeyras F, Charles HC, Krishnan KRR. A repeat proton magnetic resonance spectroscopy study in social phobia. Biol Psychiatry. 1997 Sep;42(6):419–24.

32. Phan KL, Fitzgerald DA, Cortese BM, Seraji-Bozorgzad N, Tancer ME, Moore GJ. Anterior cingulate neurochemistry in social anxiety disorder: 1H-MRS at 4???Tesla: NeuroReport. 2005 Feb;16(2):183–6.

33. Yue Q, Liu M, Nie X, Wu Q, Li J, Zhang W, et al. Quantitative 3.0T MR Spectroscopy Reveals Decreased Creatine Concentration in the Dorsolateral Prefrontal Cortex of Patients with Social Anxiety Disorder. Soriano-Mas C, editor. PLoS ONE. 2012 Oct 23;7(10):e48105.

34. Howells FM, Hattingh CJ, Syal S, Breet E, Stein DJ, Lochner C. 1H-magnetic resonance spectroscopy in social anxiety disorder. Prog Neuropsychopharmacol Biol Psychiatry. 2015 Apr;58:97–104.

35. Tükel R, Aydın K, Yüksel Ç, Ertekin E, Koyuncu A. Proton Magnetic Resonance Spectroscopy in Social Anxiety Disorder. J Neuropsychiatry Clin Neurosci. 2016 Apr;28(2):138–42.

36. Pollack MH, Jensen JE, Simon NM, Kaufman RE, Renshaw PF. High-field MRS study of GABA, glutamate and glutamine in social anxiety disorder: Response to treatment with levetiracetam. Prog Neuropsychopharmacol Biol Psychiatry. 2008 Apr;32(3):739–43.
